# Cardiorespiratory Fitness and Sleep, but not Physical Activity, are Associated with Functional Connectivity in Older Adults

**DOI:** 10.1186/s40798-024-00778-6

**Published:** 2024-10-19

**Authors:** David Wing, Bart Roelands, Julie Loebach Wetherell, Jeanne F. Nichols, Romain Meeusen, Job G. Godino, Joshua S. Shimony, Abraham Z. Snyder, Tomoyuki Nishino, Ginger E. Nicol, Guy Nagels, Lisa T. Eyler, Eric J. Lenze

**Affiliations:** 1 Herbert Wertheim School of Public Health and Human Longevity Science, University of California, San Diego, USA; 2 Exercise and Physical Activity Resource Center (EPARC), University of California, San Diego, USA; 3 Department of Psychiatry, Washington University School of Medicine, St. Louis, MO USA; 4 Mental Health Service, VA San Diego Healthcare System, San Diego, USA; 5 Department of Psychiatry, University of California, San Diego, USA; 6 Human Physiology & Sports Physiotherapy Research Group, Faculty of Physical Education and Physiotherapy, Vrije Universiteit Brussel, Brussels, Belgium; 7 Education, and Clinical Center, Desert-Pacific Mental Illness Research, San Diego Veterans Administration Healthcare System, San Diego, USA; 8 Department of Neurology, Brussels, Belgium/Center for Neurosciences (C4N), UZ Brussel, Vrije Universiteit Brussel (VUB), Brussels, Belgium; 9 Mallinckrodt Institute of Radiology, Washington University School of Medicine, St. Louis, MO USA; 10 Vrije Universiteit Brussel, Brussels, Belgium; 11 Department of Sports, Recreation, Exercise and Sciences, Community and Health Sciences, University of the Western Cape, Cape Town, South Africa

**Keywords:** Functional connectivity, Brain health, Maximal cardiovascular fitness, Successful aging, Physical activity, Body composition, Sleep quality, Sleep quantity

## Abstract

**Background:**

Aging results in changes in resting state functional connectivity within key networks associated with cognition. Cardiovascular function, physical activity, sleep, and body composition may influence these age-related changes in the brain. Better understanding these associations may help clarify mechanisms related to brain aging and guide interventional strategies to reduce these changes.

**Methods:**

In a large (n = 398) sample of healthy community dwelling older adults that were part of a larger interventional trial, we conducted cross sectional analyses of baseline data to examine the relationships between several modifiable behaviors and resting state functional connectivity within networks associated with cognition and emotional regulation. Additionally, maximal aerobic capacity, physical activity, quality of sleep, and body composition were assessed. Associations were explored both through correlation and best vs. worst group comparisons.

**Results:**

Greater cardiovascular fitness, but not larger quantity of daily physical activity, was associated with greater functional connectivity within the Default Mode (*p* = 0.008 r = 0.142) and Salience Networks (*p* = 0.005, r = 0.152). Better sleep (greater efficiency and fewer nighttime awakenings) was also associated with greater functional connectivity within multiple networks including the Default Mode, Executive Control, and Salience Networks. When the population was split into quartiles, the highest body fat group displayed higher functional connectivity in the Dorsal Attentional Network compared to the lowest body fat percentage (*p* = 0.011; 95% CI − 0.0172 to − 0.0023).

**Conclusion:**

These findings confirm and expand on previous work indicating that, in older adults, higher levels of cardiovascular fitness and better sleep quality, but not greater quantity of physical activity, total sleep time, or lower body fat percentage are associated with increased functional connectivity within key resting state networks.

**Supplementary Information:**

The online version contains supplementary material available at 10.1186/s40798-024-00778-6.

## Key Points

- Cardiovascular fitness is associated with younger/healthier brains assessed in terms of functional connectivity of key resting state networks associated with cognitive capacity and emotional regulation.
- Stronger functional connectivity is associated across multiple regions with increased total sleep quality.
- Quantity of physical activity is not associated with stronger functional connectivity in any of the regions associated with cognition although there is an association of increased connectivity in the Motor Control Network.
- Higher body fat percentage is associated with greater functional connectivity in the Dorsal Attentional Network.

## Background

Preservation of cognitive performance and emotional wellness are critical for successful aging [1, 2]. While lifestyle behaviors are considered important to slowing age-related changes and preserving brain function, relatively little is known about the physiological mechanisms underlying the associations between lifestyle behavior and brain aging. Given the worldwide increases in longevity [3] there is a need to consider methods to slow declines associated with aging and better preserve function across the lifespan.

Although certain brain structures are thought to be critical for cognition [4], they do not work in isolation. Instead, these structures are functionally connected to other structures and regions. Functional connectivity (FC) is measured by evaluating temporal correlations in spontaneous activity between widely separated regions of the brain [5]. A network can then be identified as groups of neurons that repeatedly “fire” together. The activity within, and between, networks can be observed while resting or completing mental or physical tasks [5]. Meaningful differences in FC between younger versus older adults have been found across a broad range of resting state networks, with less functional connectivity generally associated with older individuals. The bulk of the research suggests that differences are most pronounced in the Default Mode Network (DMN) [6–9], the Executive Control Network (ECN) [7] and the Salience Network (SAL) [7, 10] with lesser, but still significant differences in the Dorsal Attentional Network (DAN) [7]. In contrast, networks associated with sensory functions, including visual processing (VIS), and motor control (MOT) show minimal associations with older age [7, 8, 11].

Cardiorespiratory fitness (CRF), assessed as the amount of oxygen that the body can utilize during maximal effort (V02_max_), depends on several integrated processes including ventricular function, pulmonary sufficiency, vascular ability to accommodate and transport hemoglobin in blood, and cellular ability to accept oxygen and make it available to the mitochondria [12]. Although cardiorespiratory fitness is somewhat modifiable through training, it has been estimated that genetics contribute between 44 and 68% of variance across individuals [13, 14]. When considered in absolute terms (i.e. independent of body weight). In contrast, physical activity is the quantity of activity an individual accumulates throughout the day, often assessed in terms of intensity levels. Moderate to vigorous intensity is widely recognized as beneficial to overall health [15]. A substantial body of research indicates that both overall cardiovascular fitness and regular physical activity (PA) have protective and restorative effects on age-related cognitive decline and the development of neurodegenerative diseases. [16–22]. However, other literature indicates that both fitness and physical activity have minimal effects on BrainAge, a synthetic measure derived from the volume of 435 brain structures [23, 24] or that there are no differences in cognitive outcomes after substantial changes in fitness [25]. Furthermore, studies relating physical activity to measures of brain integrity have reported variable findings with generally small effect sizes, if relations are detected at all [26–28].

Links between CRF, PA, and functional connectivity, both within- and between-networks, have been described in several previous publications [7, 9, 29]. In particular, higher levels of CRF and PA have been associated with greater functional connectivity in regions associated with the ability to focus attention, organize sensory input, and apply memory to complete complicated muti-stage tasks [29–32]. However, other studies have reported discordant results, including no changes in FC with changes in cardiovascular fitness [33] and negative associations between FC and physical activity [34].

Being overweight or obese, estimated via the body mass index (BMI), is commonly associated with poor brain health, independently of the impact on measures of fitness (i.e., V02_max_). Specifically, higher BMI has been associated with lower volume of grey matter across several brain regions [35] and obesity, particularly central obesity, is associated with increased risk of developing Alzheimer’s Disease (AD) [36, 37]. Similarly, high BMI has been associated with reduced FC in key resting state brain networks associated with cognitive function [38, 39]. Further, recent systematic reviews of cross-sectional studies have concluded that central obesity, measured via waist circumference, is correlated with brain structural declines [40] and impaired cognition [41]. It is worth noting that the majority of included studies have relied on relatively crude measures of obesity, such as BMI and waist circumference. However, recently published studies using more sophisticated Dual X-Ray Absorptiometry (DXA) based measures of body composition have reported similar findings, with visceral adiposity being associated with brains that appear to be older than predicted based on chronological age [23, 24].

Finally, both sleep quality and quantity have exhibited equivocal associations with measures of brain health. Specifically, lower values for both sleep quantity and efficiency (a marker of sleep quality) were associated with reduced functional connectivity in the DMN in children [42], while only efficiency had the same association in adolescents [43]. Similarly, associations between poor sleep and lower functional connectivity were observed in working age, but not older adults [44]. Other studies have found that, in older adults, poor sleep predicts deteriorations in brain microarchitecture independently of other lifestyle factors [45, 46]. Additionally, in older adults, both very short (< 6 h) and very long (> 10 h) sleep duration are associated with deteriorated brain structure [47, 48] and worse performance on cognitive tests [49, 50]. Sleep related disease may also be related to neurodegeneration. Indeed, obstructive sleep apnea (OSA) is associated both with increases in blood markers associated with the development of AD [51] and with reduced FC in cognitively normal adults [52, 53] but not those with mild cognitive impairment [53].

To better understand differences in functional connectivity in sedentary older adults, we examined cross-sectional differences in FC in the DMN, ECN, SAL, DAN, MOT and VIS resting state networks (RSNs) in relation to physiological and behavioral traits. Specifically, these analyses replicate/expand on the findings of Voss and colleagues [7] by exploring the associations between fitness and FC and physical activity and FC using a somewhat larger, and similarly well characterized population of older adults. Further, we extend those findings by also examining associations between body composition and sleep respectively with FC in these key networks. Although there is some collinearity between some of these behavioral characteristics, we believe that they can be explored both independently and collectively, as was done for the closely associated physical activity and fitness by Voss et al. [7]. Additionally, to our knowledge, all these lifestyle factors have not been objectively characterized in the same healthy older adult population. We hypothesized that superior cardiorespiratory fitness, as measured by V02_max_, is associated with greater FC in the DMN, ECN, and SAL, and that higher levels of physical activity are associated with greater resting state activity in the MOT network. Additionally, we hypothesized that higher levels of adiposity, particularly visceral adipose tissue (VAT), would be associated with lower levels of functional connectivity in the DMN, ECN and SAL. Finally, we hypothesized that more sleep, both in terms of overall minutes of sleep and sleep efficiency, would be associated with increased functional connectivity in those regions.

## Methods

*Participants*: These analyses were conducted on data gathered during the baseline measurement of a longitudinal intervention set in two urban areas and approved by the Institutional Review Board at both The University of California, San Diego (UCSD), and Washington University in St. Louis (WUSTL). All participants provided informed consent to participate. The larger group of 607 older adults has been extensively described elsewhere [25, 54]. Key details meaningful to the current analyses include that participants were between ages 65 and 84 and had self-reported cognitive complaints but were free from assessed cognitive impairment (defined as < 11 on Short Blessed Test [55]) or diagnosed neurodegenerative disease. Additionally, participants were excluded if they were currently using glucocorticoid or diabetes medication, were too physically active (defined as > 60 min/week of moderate to vigorous exercise any week within the last 6 months), reported alcohol or substance abuse within the previous six months, or reported having a disease or condition that would make it impossible to participate in the (exercise and/or mindfulness) intervention(s). Additionally, participants completed a maximal exercise test (see below) during their measurement visit and were excluded if they exhibited substantial cardiac arrhythmia or ischemia.

### Physical Measures (GXT, DXA, Accelerometery)

*Graded Exercise Testing (GXT)*: The majority of participants completed a GXT using a treadmill (Quinton QStress, Cardiac Science, Chelmsford, Mass) with a substantially smaller number (< 10%) who were unable to walk without holding onto the treadmill handrails using an electronically braked cycle ergometer (LODE Excalibur, Netherlands) to volitional maximal exertion. The testing protocol has been described previously [23, 54]. In brief, under the supervision of a physician, participants warmed-up for three to five minutes and then intensity was increased at a level equal to approximately 0.6 change in the metabolic equivalents of task (METS) per two-minute stage with active motivation from study staff (n = 2 minimum offering ongoing and enthusiastic verbal encouragement). Exercise grew progressively harder until the physician ended the test based upon potentially dangerous changes in the ECG reading or an extreme hypertensive response (SBP > 220 or DBP > 110), or the participant indicated that they were unwilling/unable to continue. Individuals were excluded if they did not reach a minimum of 85% of their age predicted heart rate maximum (220-age) or testing was stopped by the physician prior to volitional fatigue. Maximal capacity was calculated in METs using the American College of Sports Medicine’s algorithm [56] designed for walking or cycling as appropriate based upon testing methodology. Based on the presumed linear relationship in oxygen consumption while coming to a metabolic steady state in response to a new workload, partial stages were scored in 30 s increments using the formula METs _last_completed_stage_ + 0.25* METs _difference_between_stages_* number of 30 s increments completed in the new stage.

*Dual X-Ray Absorptiometry (DXA) to estimate body composition*: A GE Lunar Prodigy at one location and an iDXA (both GE/Lunar, Madison, WI) at the other were used to estimate segmental body composition and provide absolute values (measured in grams or kilograms) for fat and lean tissue and bone mass for the arms and legs and trunk as well as secondary height-dependent regions titled android (centered on the abdomen) and gynoid (centered on the upper thighs). Visceral fat values were also derived from the android region. This method of estimating visceral fat has had good agreement compared to 3-D imaging techniques in both men and women [57, 58].

Participants were positioned in line with best practice recommendations [59] and external artifacts were removed whenever possible. For participants who were larger than the available scan field, a “hemi-scan” was acquired by scanning only the right side of the body and replicating those values for the “missing” limb.

*Accelerometry*:* Measurement of physical activity*: Participants were asked to maintain their normal behaviors during a 10 day period during which they were equipped with an Actigraph GT9X + Link (ActiGraph Inc, Pensacola, FL) deployed in line with best practice recommendations [60–63] worn on the participant’s non-dominant wrist continuously except when engaging in water-based activities like swimming or bathing. This tri-axial accelerometer has been shown to be both valid and reliable across the age span [60, 64, 65]. After 10 days of deployment, devices were recovered and data were downloaded and screened for completeness and potential device malfunction in line with established practice [60, 62, 66]. Data processing included applying a screening algorithm to detect non-wear [67, 68] and aggregating raw data into “counts per minute” in the x, y and z axes independently using Actilife (Actigraph’s proprietary software). Vector magnitude was calculated using the square root of the sum of the squares of the three axes to incorporate intensity, frequency, and duration of movement. This metric, henceforth referred to as Vector Magnitude Counts Per Minute (VM CPM) has been recommended for use in assessing physical activity during a 24-h wear period [69] particularly when the device has been deployed at the wrist.

*Accelerometry*:* Measurement of sleep*: The same device was used to calculate sleep time in total minutes, wake after sleep onset (WASO) both in terms of number of awakenings and number of minute awake, and sleep efficiency using an algorithm that has been validated for use in adults [70]. Participants were specifically asked to maintain their normal sleep rhythms during the wear period. The window of observation was derived from sleep journals in which participants indicated the time that they had begun trying to sleep, and the time that they first woke up in the morning (i.e. there was no effort to record incidental, or undesired, nighttime awakening). All records were manually entered and inspected by a specifically trained research assistant. If, based on visual inspection more than 50% of a period at the immediately following the “go to bed” or preceding the “first wakening” time appeared to have substantial amounts of movement indicative of poor subject record keeping, in-bed or first awakening time was adjusted to reflect the period when movement appeared to (mostly) cease. If a sleep journal was not maintained, previously validated methods were utilized to estimate the time of sleep onset and wake time [71]. Individuals with obstructive sleep apnea (OSA) were identified and sleep variables were compared against those without OSA.

### Imaging Measures

*Imaging Acquisition*: Three magnetic resonance imaging (MRI) scanners at two sites were used to acquire resting state functional MRI (rs-fMRI) data (UCSD: GE MR750 3 T scanner (GE, Milwaukee, WI) with an 8-channel head coil; WUSTL: Siemens 3 T Trio and 3 T Prisma-FIT (Erlangen, Germany) with a 20-channel head coil. T1-weighted (T1w) and T2-weighted (T2w) structural scans were performed for purposes of image registration and radiological screening of the participants (UCSD: T1 MPRAGE, TE = 3.036 ms, TI = 1.000 ms, 1.0 mm^3^ voxels; T2 CUBE, TR = 3.300 ms, TE = 73.37 ms, 1.0 mm^3^ voxels; WUSTL: T1 MPRAGE, TR = 2.400 ms, TE = 3.16 ms, TI = 1.000 ms, 1 mm^3^ voxels; T2 SPACE, TR = 3.200 ms, TE = 458 ms, 1 mm^3^ voxels). Four rs-fMRI scans (140 frames per run) were acquired per subject using a multi-echo sequence (UCSD: TR = 2.740 ms, TE = 14.8, 28.4, 42, 55.6 ms; 4.0 mm^3^ voxels; WUSTL: TR = 2.960 ms, TE = 15, 31.3, 47.6, 63.9 ms; 4.0 mm^3^ voxels) with a total run time of 6.4 min (UCSD) or 6.9 min (WUSTL) per run or 25.6 and 27.6 total scan minutes respectively. Gradient echo field maps were acquired for later use in correction of susceptibility-related image distortion. During fMRI, participants were shown a neutral video without audio (e.g., nature scenes) synchronized to the start of each run and were asked to stay awake without engaging in any sort of meditation.

*Image Preprocessing*: fMRI data processing largely followed previously described methods [72] which makes use of several FSL modules [73]. Briefly, rigid body motion correction both within- and across-runs was computed on data summed over all echos. Slice timing correction was applied to each echo. Bias field inhomogeneities were corrected using the FAST module in FSL [74]. Atlas transformation was computed by composition of transforms (individual frame → frame average → T2w → T1w → atlas representative target). T1w → atlas registration was computed with FSL FNIRT. Three atlas representative targets, all representing the 711-2B version of Talairach space, had been previously prepared for each of the scanners to accommodate scanner-specific differences in T1w contrast. Final resampling of the fMRI data in 3 mm^3^ voxel 711-2B space was accomplished in one step incorporating distortion correction, previously computed bias field correction, and the composition of all spatial transforms.

The multi-echo data were then modeled according to standard theory [75] fitting the four echoes to a monoexponential model S_t = S_0t⋅exp(− R_2t^⋆⋅TE_k:, where indexes frame, indexes echo, and is reconstructed intensity extrapolated to echo time 0. Frame-to-frame variation in was suppressed by averaging over the whole run and the fMRI data were modeled at a TE of 30 ms according to S_t = (S_0) ®⋅exp(− R_2t^⋆⋅30 ms). The modeled data in each run then were intensity normalized (one multiplicative scalar applied to all voxels and frames) to achieve an intensity mode value of 1000.

Denoising was effected on the fMRI data virtually concatenated across the 4 runs. Frame censoring was computed on the basis of DVARS [76], with the criterion adjusted to compensate for baseline variability using a previously described method based on fitting the distribution DVARS values to a gamma function [77]. Subsequent steps ignored all censored frames. The data were denoised using a CompCor-like scheme with regressors derived from motion correction temporally filtered to suppress respiration-related factitious head motion [78], white matter, ventricles, extra-axial cerebral spinal fluid (CSF), and the whole brain global signal [79]. Image derived regressors were based on tissue class segmentations computed by FreeSurfer 6.0.0 [80]. Additional denoising included bandpass temporal filtering retaining frequencies in the range 0.01–0.1 Hz and spatial filtering (Gaussian blur of 6 mm in each cardinal direction). Finally, the (scanner-specific) response evoked by the movie was averaged over all participants and subtracted from each individual’s data.

**Fig. 1:**
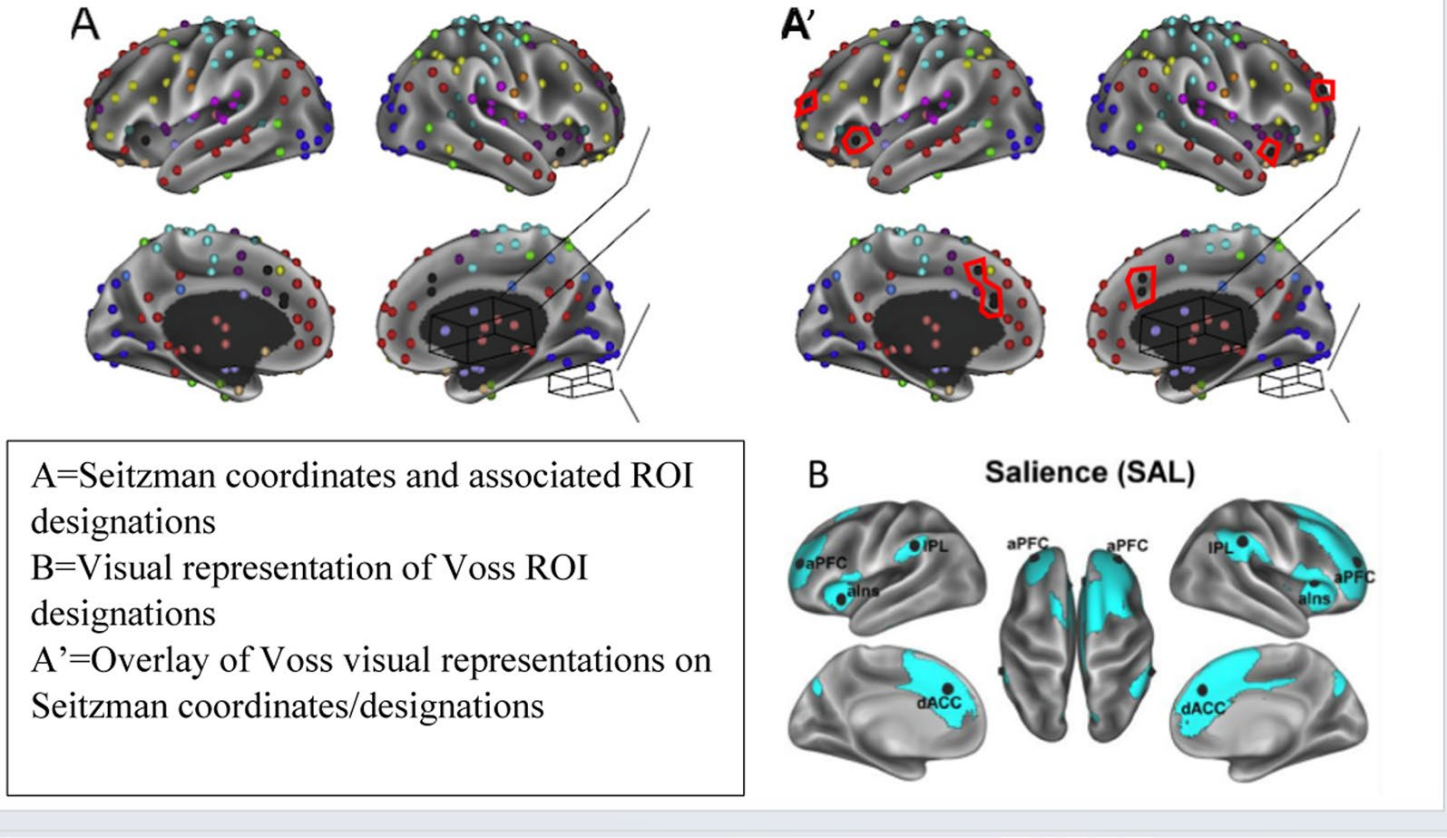
Seitzman and Voss coordinates and ROI’s—salience network. Seitzman ROI’s as designated by different colors on the top left (**A**) and Voss ROI’s are designated on the bottom right (**B**). The overlay areas of the two are shown on the top right (**A’**). Note: Visual representation of overlay between other networks of interest are available in supplementary materials

*ROI/Network Creation*: Relevant voxel locations were initially identified using the Big Brain 300 parcellation described by Seitzman and colleagues [81] excluding subcortical and cerebellar RIO’s. In an effort to confirm/replicate earlier findings we projected the regions described by Voss and colleagues [7] onto the Seitzman ROI’s. Thus, three networks were defined; the salience (SAL), motor control (MOT), and visual (VIS) networks. Two additional networks described by Voss et al. [7] the default mode network (DMN) and the dorsal attention network (DAN) were substantially different when compared to Seitzman and colleagues parcellation [81]. Accordingly, we utilized both with labels DMN and DAN for Voss defined regions and BSDMN and BSDAN for Seitzman. Finally, because Seitzman and colleagues did not identify an executive control network (ECN) we utilized the Voss visual representations and included voxels identified as frontoparietal and DMN within the Sietzman designations. The voxel locations and network designates are included in the supplementary materials. A visual representation of Seitzman-Voss mapping for SAL is shown in Fig. 1 below, and mapping for all five examined networks are shown in supplementary Figs. 1 through 5. The average correlation value, defined as the average correlation across the ROI x ROI pairs within each network was calculated and assessed as the within network connectivity value for subsequent analyses.

### Statistics

*Statistical Analysis*: SPSS version 28 was used to conduct all statistical analyses. Participants were excluded from any analysis for which they had missing values. Descriptive statistics (proportions, mean ± standard deviation) were used to characterize the population, and t-tests were used to compare differences by study site and sex. Correlations between physiological/behavioral variables and FC with age were examined with Pearson product-moment correlations. In addition, inter-relationships among the physiological/behavioral variables and among the networks of interest were evaluated while controlling for chronological age, sex, location, and years of education with Bonferroni adjustments applied to correct for multiple comparisons.

For examination of the relationship between physiological/behavioral variables and the regional FC variables, we used multiple linear regressionto create unstandardized residual values for the resting state functional networks after controlling for sex, chronological age, years of education, and location. Further, unstandardized residual values were created for physiological/behavioral variables using multiple linear regression that controlled for covariates. Specifically, we controlled for chronological age, sex, and location for all variables. Years of education was not included as a covariate as it was not hypothesized to be associated with any of these variables, whereas including it was appropriate when considering FC. Additionally, based on the known association between physical activity and fitness, in order to create appropriate residuals we included physical activity as a covariate for analyses involving fitness. With this association in mind, and the known association between fitness and body composition, fitness was included as a covariate for creating physical activity and body composition unstandardized residuals. Finally, we also included both sleep efficiency and total sleep time as a covariate for creating both fitness and PA residuals. Similarly, fitness and PA were included as covariates for creating all sleep residuals. In both directions, this decision is based on the growing evidence regarding a dynamic interplay between these sleep, fitness, and activity variables in relation to brain health [82, 83]. The final unstandardized residuals then represent the individual differences in the variable of interest after variance from the covariates has been accounted for.

We then conducted continuous linear regression using the unstandardized residuals of the behavioral variables of interest as the independent variable and the unstandardized residuals of the FC networks of interest as the dependent variable. Additionally, in line with the work of Voss and colleagues [7] on physical activity and fitness, we divided participants into the top and bottom 25% of the cohort based upon the unstandardized residual values for each physiological/lifestyle variable of interest to further evaluate the potential differences in unstandardized FC residuals at key networks as a function of fitness (and activity, body composition, and sleep each considered independently). We then compared these groups with independent samples t-tests following the hypothesis that the “best” 25% would have greater FC than the “worst” 25% using Bonferroni adjustments to correct for multiple comparisons.

## Results

### Population Descriptives

A total of 398 participants (195 San Diego, 203 WUSTL) were included in the overall analyses. Of these 398, forty-five participants who did not continue to maximal effort (determined as not reaching 85% of age predicted heart rate max or having the study physician end the test prior to volitional fatigue) were excluded from the analysis of fitness (n = 353 for fitness measures). Five participants did not have enough night-time accelerometer wear and three insufficient daytime wear for inclusion (4 night or days respectively) leaving 393 participants included in analyses regarding sleep and 395 in daily physical activity. Sleep variables for individuals with OSA were not significantly different from those without (*p* range from 0.228 to 0.982) so all sleep data was considered for the entire population. A graphical representation of sample size with reasons for exclusion is included in the supplementary materials.

The sample self-identified largely as white (n = 314, 79%) with a smaller percentage identifying as black (n = 37, 9%), white with Hispanic ethnicity (n = 24, 6%) and Asian (13, 3%) with the remaining 10 individuals refusing to answer or indicating that they identified with multiple racial/ethnic categories. The sample was predominantly female (78%), and females in the sample population were younger than males. As expected, based on population level statistics, women had lower maximal cardiovascular fitness, higher overall body fat percentage and lower lean body mass. However, men had greater VAT and less overall physical activity as measured in VMCPM. Finally, although FC across the majority of the networks was not significantly different by gender, women had greater connectivity in the DMN. Variables with statistically significant difference, along with confidence intervals are included in supplementary materials Table 1.

**Table 1:** Sample characteristics by intervention location

	Total group	UCSD	WUSTL	*P* =
% Female	78	79	73	0.610
Age (yrs)	71.3 (4.7)	71.6 (4.7)	71.1 (4.8)	0.246
Education (yrs)	16.2 (2.2)	16.2 (2)	16.2 (2.3)	0.791
Maximal cardiovascular fitness (METS)	7.1 (1.8)	6.6(1.5)	7.6(1.9)	**< .001**
Body fat (%)	40.2 (7.4)	40.3 (7.5)	40.2 (7.4)	0.902
Lean tissue (g)	42,786.2 (8486)	42,556.5 (8870)	43,006.8 (8115.9)	0.597
Visceral adipose tissue (g)	1257.3 (866.9)	1276.3 (871.6)	1239.2 (864.1)	0.671
Sleep efficiency (%)	84.5 (6.3)	84.2 (6.2)	84.8 (6.4)	0.317
Total sleep time (min)	387.9 (51.8)	385.4 (52.1)	390.3 (51.5)	0.348
Nightly awake time (min)	71.3 (30.9)	72.8 (31.4)	69.8 (30.4)	0.341
Nighly awakenings (n)	18.4 (6)	18.7 (6.2)	18 (5.9)	0.218
Total movement (VM CPM)	1956.8 (502.9)	2005 (512)	1910.7 (490.9)	0.062
DMN connectivity	0.225 (0.066)	0.251 (0.063)	0.199 (0.058)	**< .001**
ECN connectivity	0.084 (0.032)	0.101 (0.031)	0.067 (0.024)	**< .001**
DAN connectivity	0.106 (0.033)	0.123 (0.03)	0.090 (0.027)	**< .001**
SAL connectivity	0.339 (0.103)	0.384 (0.092)	0.295 (0.095)	**< .001**
MOT connectivity	0.272 (0.112)	0.319 (0.109)	0.228 (0.095)	**< .001**
VIS connectivity	0.204 (0.068)	0.235 (0.07)	0.174 (0.051)	**< .001**
BSDMN connectivity	0.131 (0.045)	0.151 (0.043)	0.112 (0.039)	**< .001**
BSDAN connectivity	0.176 (0.062)	0.19 (0.061)	0.162 (0.061)	**< .001**

Bold values indicate statistical significance

*Yrs* years; *METS* metabolic equivilant of task; *VM* vector magnitude; *CPM* counts per minute; *g* grams; *min* minutes; *DMN* default mode network; *ECN* executive control network; *DAN* dorsal attentional network; *SAL* salience network; *MOT* motor control network; *VIS* visual network; *BS* Ben Sietzman defined network

### Locational Differences

There was a significant difference between the locations for participants’ maximal cardiovascular fitness and connectivity with WUSTL having a population with higher fitness and less connectivity. Means, standard deviations and p values for difference by location for all demographic, physiological, and connectivity values are shown in Table 1. Mean differences and confidence intervals of the difference for variables with significant differences for both location and sex are shown in supplementary materials Table 1.

### Correlations

Significant correlations between age and the SAL, VIS and BSDMN networks were observed (*p* = < 0.001, 0.026 and 0.016 respectively). As such, age was included as a covariate in other correlational analyses. After controlling for age, sex, and location all resting state functional networks had significant positive correlation (*p* = < 0.001–0.005) except for between VIS and BSDAN (*p* = 0.763).

**Fig. 2:**
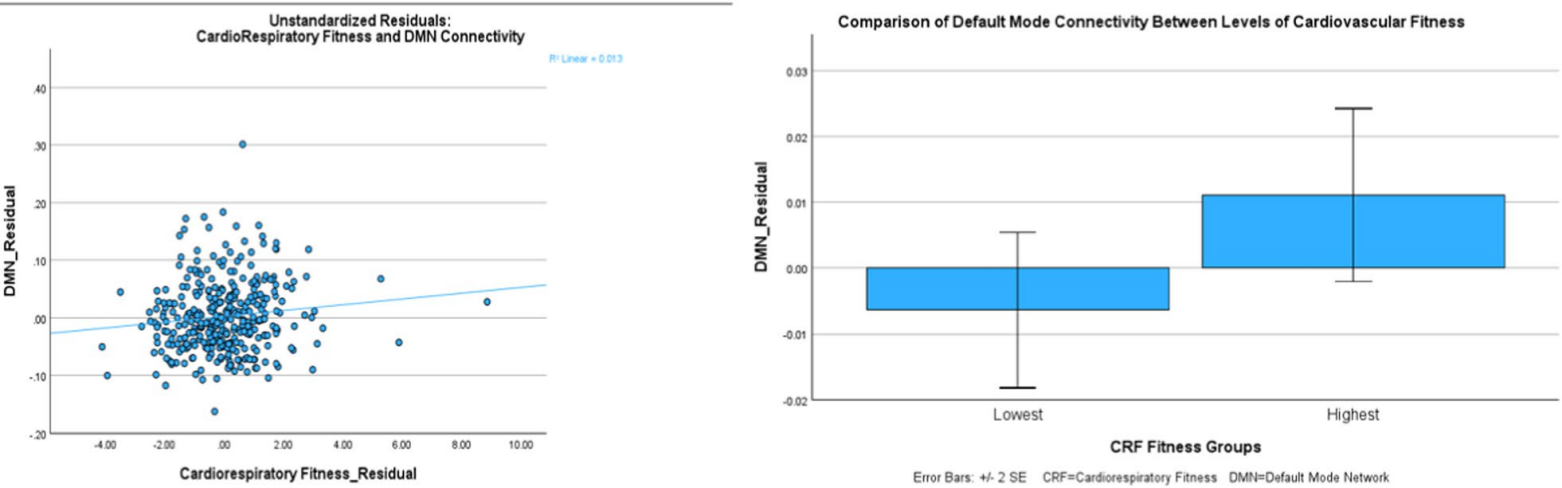
Cardiorespiratory fitness and DMN connectivity shown both continuously and split into best and worst quartile. Left-hand graph = Continuous comparison of unstandardized residuals of the CRF and DMN connectivity (*p* = 0.040). Right-hand graph = T-test based comparison of the best and worst CRF residual groups with DMN connectivity (*p* = 0.049). CRF = Cardiorespiratory Fitness; DMN = Default Mode Network

Similarly, many physiological variables were associated. Specifically, cardiorespiratory fitness was associated with total physical activity (*p* < 0.001 r = 0.374) and all metrics of body composition (*p* range < 0.001 and = 0.024). Additionally, all metrics of body composition were associated with each other (*p* < 0.001 for all). All sleep-based variables were also significantly associated (*p* < 0.001 for all). In addition to the relationship to CRF mentioned above, total daily physical activity was negatively associated with metrics of body fatness (i.e. more movement = less fatness). Further, PA was negatively associated with sleep efficiency and time, and positively associated with wake after sleep time (i.e. more movement = less sleep time and lower sleep quality). A scatterplot showing the significant associations between fitness and DMN is shown in Fig. 2 below. All other correlation matrix tables and scatterplots for both network to network associations and associations between physiological variables are provided in the supplementary materials.

Cardiorespiratory fitness was correlated with DMN (*p* = 0.008 r = 0.142), SAL (*p* = 0.005, r = 0.152) and BSDMN (*p* = 0.008 r = 0.143) but not with ECN, DAN, MOT, VIS, or BSDAN (*p* range = 0.248–0.982). Total physical activity was not significantly associated with greater MOT connectivity (*p* = 0.060; r = 0.124), or any other network (*p* range = 0.156–0.862).

When exploring associations between FC and body composition, the only significant associations were between percent body fat and the VIS (*p* = 0.03, r = 0.117), DMN (*p* = 0.05; r = 0.105), and DAN (*p* = 0.047; r = 0.107) networks.

Greater sleep efficiency was associated with greater connectivity in the SAL (*p* = 0.007; r = 0.137) and BSDMN (*p* = 0.016; r = 0.122) networks. Total sleep time was inversely associated with ECN, MOT and VIS connectivity (*p* = 0.016, 0.025 and 0.027 and r = − 0.121, − 0.114 and − 0.112 respectively). Total amount of time awake during (attempted) sleep periods was negatively associated with multiple networks including SAL (*p* = < 0.001, r = − 0.18), MOT (*p* = 0.007; r = − 0.136), BSDMN (*p* = 0.038; r = − 0.105) and BSDAN (*p* = 0.043; r = − 0.102). The number of times awakening during a sleep period (regardless of total length of time awake) was negatively associated with ECN (*p* = 0.001; r = − 0.162) and MOT (*p* = 0.013; r = − 0.126).

### Results of Residual Based Linear Regressions and Comparisons of Best vs. Worst Quartiles

In an effort to provide a visual representation of the potential ways that difference can be observed with these analyses, Fig. 2 shows graphical representations of the data shown both continuously and grouped into best v. worst groupings between CRF and DMN connectivity.

Linear regression using unstandardized residuals for all variable categories yielded significant results related to multiple behavioral variables and the SAL and MOT networks, with a smaller number showing significant predictive association with the DMN or ECN. Table 2 below provides details regarding the model fit, and beta values for continuous regressions that achieved statistical significance after correction. For those interested in greater detail, the model fit, standard error, and beta values for all of the variable combinations are available in the supplementary materials.

**Table 2:** Linear regressions: unstandardized continuous variables

Behavioral variable	Functional network	Model summary R2		Unstandardized β	SE	*p* value
CRF (METS)	DMN	0.012	Constant	0.002	0.003	0.040
			CRF (METS)	0.005	0.002	
Body fat (%)	SAL	0.011	Constant	1.909E-16	0.005	0.035
			Body fat (%)	− 0.002	0.001	
VAT (g)	SAL	0.012	Constant	0.001	0.005	0.028
			VAT (g)	− 1.36E-05	0	
PA (VM_CPM)	MOT	0.023	Constant	0.001	0.005	0.005
			PA (VM_CPM)	3.44E-05	0	
Sleep quality (%)	SAL	0.021	Constant	0.000	0.005	0.007
			Sleep quality (%)	0.002	0.001	
Sleep quality (%)	MOT	0.014	Constant	0.001	0.005	0.026
			Sleep quality (%)	0.002	0.001	
Sleep time (min)	ECN	0.025	Constant	0.001	0.001	0.003
			Sleep time (min)	− 8.69E-05	0	
Sleep time (min)	VIS	0.017	Constant	− 7.01E-05	0.003	0.015
			Sleep time (min)	0.000	0	
Wake time (min)	SAL	0.033	Constant	0.000	0.005	< .001
			Wake time (min)	− 0.001	0	
Wake time (min)	MOT	0.025	Constant	0.001	0.005	0.003
			Wake time (min)	− 0.001	0	
Wake number (n)	ECN	0.018	Constant	0.001	0.001	0.011
			Wake number (n)	− 0.001	0	
Wake number (n)	SAL	0.014	Constant	0.000	0.005	0.030
			Wake number (n)	− 0.002	0.001	
Wake number (n)	MOT	0.022	Constant	0.001	0.005	0.005
			Wake number (n)	− 0.003	0.001	

*CRF* cardiorespiratory fitness; *CPM* counts per minute; *DMN* default mode network; *ECN* executive control network; *g* grams; *min* minutes; *MET* metabolic equivilant of task; *MOT* motor control network; *SAL* salience network; *VIS* visual network; *VM* vector magnitude

Modest predictive associations exist indicating that CRF significantly positively contributes to DMN connectivity (*p* = 0.040). Additionally, body fatness contributes significantly to reduced connectivity in the SAL network. This is true both in terms of total body fat percentage, and visceral adiposity (*p* = 0.035 and 0.028 respectively). Greater amounts of total daily physical activity are associated with increased connectivity within the MOT network (*p* = 0.005). Finally, sleep quality expressed both as percent of time in bed spent asleep and number of nightly awakenings are predictive of connectivity across multiple networks in a manner in which better sleep is associated with greater connectivity.

Results of the t-tests comparing the best versus worst quartiles within key metrics of interest are shown in Table 3. The most fit participants had significantly higher connectivity in the DMN, SAL and BSDMN compared with the least fit (*p* = 0.049, 0.015 and 0.023 respectively). Additionally, the quartile with the highest body fat percentage had higher connectivity in the DAN (*p* = 0.011) with no differences in any networks observed for lean tissue or VAT (*p* > 0.05). Individuals with the most overall physical activity had higher MOT connectivity (*p* = 0.001). The quartile with the best sleep efficiency showed greater connectivity in the DAN (*p* = 0.050), SAL (*p* = 0.003), BSDMN (*p* = 0.032) and MOT (0.024) The quartile with the lowest amount of time awake during sleep had greater connectivity in the SAL (*p* = < 0.001), and MOT (*p* = 0.004) networks while those with the fewest number of times awakening showed greater connectivity only in the BSDAN (*p* = 0.020).

**Table 3:** T-tests and confidence intervals comparing first (top 25%) vs fourth (bottom 25%) quartile for each behavioral variable

	Fitness	Body fat	VAT	Physical activity	Sleep quality	Sleep time	Wake time	Wake number
*p* =	(METS)	(%)	(kg)	(CPM)	(%)	(min)	(min)	(n)
DMN	**0.049**	0.811	0.839	0.564	0.366	0.146	0.419	0.257
ECN	0.250	0.120	0.260	0.192	0.251	0.072	0.169	0.051
DAN	0.469	**0.011**	0.123	0.322	**0.050**	0.304	0.189	0.449
SAL	**0.015**	0.082	0.094	0.954	**0.003**	0.878	** < .001**	0.083
BSDMN	**0.023**	0.204	0.423	0.499	**0.032**	0.066	0.121	0.199
BSDAN	0.935	0.890	0.531	0.148	0.077	0.691	0.199	**0.020**
Mot	0.753	0.757	0.823	**0.001**	**0.024**	0.209	**0.004**	0.287
Vis	0.595	0.186	0.432	0.400	0.725	0.123	0.862	0.442
*95% Confidence Intervals*								
DMN	**− 0.0349 to − 0.0001**	− 0.0133 to 0.0169	− 0.0167 to 0.0136	− 0.0229 to 0.0125	− 0.0271 to 0.0101	− 0.0315 to 0.0047	− 0.0106 to 0.0253	− 0.012 to 0.0445
ECN	− 0.0035 to 0.0133	− 0.0126 to 0.0015	− 0.012 to 0.0033	− 0.0026 to 0.0129	− 0.0138 to 0.0036	− 0.0007 to 0.0159	− 0.0026 to 0.0148	0 to 0.0288
DAN	− 0.0119 to 0.0055	**− 0.0172 to − 0.0023**	− 0.0131 to 0.0016	− 0.0136 to 0.0045	**− 0.0173 to − 0.001**	− 0.013 to 0.0041	− 0.0028 to 0.0139	− 0.0088 to 0.0197
SAL	**− 0.0589 to − 0.0064**	− 0.0027 to 0.0459	− 0.0035 to 0.0445	− 0.0297 to 0.0281	**− 0.0702 to − 0.0152**	− 0.0297 to 0.0254	**0.0205 to 0.0747**	− 0.0054 to 0.0872
BSDMN	**− 0.0261 to − 0.0019**	− 0.0034 to 0.0158	− 0.0061 to 0.0145	− 0.0162 to 0.0079	**− 0.026 to − 0.0011**	− 0.024 to 0.0008	− 0.0024 to 0.0203	− 0.0064 to 0.0301
BSDAN	− 0.0196 to 0.018	− 0.0158 to 0.0182	− 0.0218 to 0.0113	− 0.0321 to 0.0049	− 0.0357 to 0.0018	− 0.0225 to 0.015	− 0.0061 to 0.0291	**0.0057 to 0.0646**
Mot	− 0.0273 to 0.0377	− 0.035 to 0.0255	− 0.0247 to 0.031	**− 0.0763 to − 0.0193**	**− 0.0673 to − 0.0047**	− 0.011 to 0.0498	**0.0142 to 0.075**	− 0.0244 to 0.0817
Vis	− 0.0221 to 0.0127	− 0.0276 to 0.0054	− 0.0226 to 0.0097	− 0.0103 to 0.0256	− 0.0162 to 0.0232	− 0.0035 to 0.0293	− 0.0212 to 0.0178	− 0.0186 to 0.0423

Bold values indicate statistical significance

Note, desirability of first vs. fourth quartile varies according to metric

The lowest quartile is presumed to be healthier for body fat, VAT, Wake-time and Wake number

The highest quartiles is presumed to be healthier for fitness, PA, Sleep Quality, and Sleep Time

All variables controlled for sex, age, location

Fitness variable controlled for physical activity and sleep time and sleep efficiency

Physical activity variable controlled for fitness, sleep time, and sleep efficiency

Sleep variables controlled for physical activity, and fitness

*METS* metabolic equivalent of task; *VM* vector magnitude; *CPM* counts per minute; *kg* kilograms; *min* minutes; *DMN* default mode network; *ECN* executive control network; *DAN* dorsal attentional network; *SAL* salience network; *MOT* motor control network; *VIS* visual network; *BS* Ben Sietzman defined network

It is worth noting that the choices made for the spatial location of component ROIs within key networks have some impact on the analyses findings and resulting conclusions. In general, both correlative and comparative significance (or non-significance) was observed in both the Seitzman and Voss defined DMN and DAN concurrently. However, that was not always the case (see Table 2 for differences in significance by definitional region). We focused on the Voss defined regions to better position our conclusions with existing literature regarding fitness, body composition, activity, and sleep.

## Discussion

### Overall Findings

The present data suggest that some, but not all, of the lifestyle-based behaviors assessed here are associated with differences in FC within key brain networks in a population of sedentary older adults. More specifically, better fitness and sleep efficiency, but not greater daily physical activity or total sleep quantity, were associated with greater functional connectivity in the DMN and SAL networks. Further, good sleep quality, defined as fewer nightly awakenings, was associated with greater connectivity in the SAL network. Although less powerful than the associations seen between FC and sleep efficiency and fitness, we found that, contrary to expectations, higher percentage body fat was associated with greater FC in the DAN, and that less total sleep time was associated with greater FC in the ECN. Although causality cannot be determined from these cross-sectional analyses, these results further inform the associations between lifestyle factors and brain aging and provide additional insight into possible mechanisms underlying age related differences in brain physiology.

### Cardiorespiratory Fitness

We found relations between greater aerobic fitness and stronger FC in the DMN and SAL networks. Interestingly, we did not find a predictive association when CRF and the SAL network are considered continuously, but did find significant differences in this network when comparing “best” and “worst”. This suggests that, at least for some networks, there is a threshold above which fitness is protective. These data strengthen, through replication, previous results [7, 84, 85] indicating that the most fit individuals had stronger connectivity in the DMN and SAL networks than the least fit, and that greater connectivity in these regions is associated with “younger” brains [7]. The significance of these associations is further strengthened by the fact that the mean difference in connectivity was greater than 7% in these networks relative to the FC mean over the total population.

These observed associations are consistent with the cardiovascular fitness hypothesis, which postulates that to meaningfully impact health, physical activity must be at an intensity sufficient to improve fitness [7, 84, 85], and thus light physical activity is unlikely to be beneficial. There are several mechanisms that could possibly contribute to the observed associations. Potentially the most widely studied is the increased/improved cerebral blood flow related to greater microvasculature associated with better fitness. [86]. Others have suggested that individuals with higher CRF may have actually have lower blood flow needs within key regions associated with the DMN and SAL [87]. Alternatively, given that higher levels of fitness are often associated with less systemic inflammation ([88], it may be that greater CRF is also protective against neuroinflammation and its negative downstream effects [89]. Regardless of mechanism, given the known associations between the ability to reflect/learn and FC in the DMN [90, 91] and to regulate emotions and FC in the SAL network [92, 93], aerobic capacity may be important to successful aging of the brain in addition to its role in cardiovascular and metabolic health.

### Physical Activity

In contrast, we did not find that daily quantity of physical activity was significantly associated with FC in cognitively important networks. Although wrist worn devices limited our ability to differentiate activity intensity, Bassett and colleagues [69] found that this metric was sufficient to identify meaningful differences in health across the NHANES population and recommended it as a metric to accurately measure total daily physical activity. Our results contrast with previous results which have found increased FC in the DMN, SAL, and ECN and with greater amounts of self-reported physical activity [94] and in the DMN with greater accelerometer measured activity [95, 96]. It is worth noting that none of these papers included/controlled for measures of fitness or sleep in their analyses, which may help to explain the discordant findings. Additionally, and in line with the cardiovascular fitness hypothesis introduced earlier it is likely that not enough of our (exclusively sedentary) population had a sufficiently large amount of activity (particularly moderate to vigorous activity) to detect meaningful differences in FC that might occur with more intense activity. However, the present results do further validate prior [7] findings that differences in FC attributable to cardiovascular fitness are independent of regular physical activity.

The observed association between all day physical activity and increased FC in the MOT network is intuitively reasonable. Although there is ample literature concerning changes in motor performance with age [97, 98] and exercise-based interventions [98, 99], there is little scholarship on the links between exercise/physical activity and MOT network functional connectivity in healthy human populations. In one of the few extant studies, sedentary youth had significantly higher MOT connectivity at rest compared to age-matched endurance runners [100]. In clinical populations, greater MOT connectivity has been observed in stroke survivors compared to healthy controls [100, 101]. Studies in both rats [102] and humans [103] following stroke suggest that these effects may reflect new neural pathways in the MOT network to compensate for lesions of the primary motor tracts. This, along with findings suggesting that older age is not associated with differences in the MOT network [7] suggest that increased resting state functional connectivity may not always represent a positive biomarker, depending on brain region/network and clinical status of the participant.

### Body Composition

The present sample indicates minimal associations between body composition and FC. When viewed continuously, there does appear to be some association with higher body fat predicting reduced connectivity. However, these contributions are not sufficiently robust to establish a significant difference between the most and least fat quartiles in this relatively large sample. This conflicts with previous results by Kullman et al. [104] who found significantly increased connectivity with increasing BMI in a continuous fashion and with other group based work that showed increased functional connectivity in the SAL network in an obese compared with non-obese population [38]. In contrast, although not significantly associated when viewed continuously (*p* = 0.061, see supplementary materials) when top vs. bottom quartiles were examined, we found that the highest fat quartile had significantly greater connectivity in the DAN, a network associated with attentional focus [98, 99]. Although minimal declines in DAN FC have been observed with advancing age [7], significantly weaker FC has been observed both in children [105] and adults with obesity [104] Thus, the current findings do not replicate certain prior results. This discrepancy could reflect population differences, as the current study sample consisted entirely of older adults with approximately one third of women, and just over half of men having body fat percentages qualifying them as obese using gender and age specific cut points derived from DXA that correspond with the traditional BMI values designating obesity. Further, it may be that the presence of additional (non-visceral) body fat is protective in older age in a way that it is not for younger adults. This has been shown in a systematic review looking at all-cause mortality and morbidity where older adults that were overweight or mildly obese had the lowest mortality [106]. The advantages of (slightly) higher non-visceral body fat may translate to brain health as well, as the associations between higher body fat and leptin production, and the associations between increasing age and decreased leptin production are both well documented [107, 108]. Although far from conclusive, there is some evidence suggesting that maintaining normal levels of leptin into older age may help to preserve memory and attentional focus [107].

### Sleep

In agreement with our a priori hypothesis, better sleep, defined as more minutes of total sleep, greater sleep efficiency, and less nightly wakeful periods, was positively associated with FC across multiple networks. This is true of sleep quality, defined as greater efficiency and less time awake during the attempted sleep period, when the data are viewed both continuously and in “best” vs “worst” quartiles. In contrast, sleep quantity (i.e. total sleep time) was only associated with ECN connectivity when viewed continuously. Although some literature suggests that more minutes of sleep minutes of sleep are associated with greater FC, and improved cognitive outcomes, particularly in working age adults [44, 109, 110] in this instance, the contribution of sleep minutes to ECN connectivity was not sufficient to establish a significant difference between the best and worst sleepers.

Greater sleep efficiency was associated with greater FC in both the SAL and MOT network, while longer amount of time awake was associated with weaker FC in the SAL and MOT networks. These findings agree with prior findings in children [42] and adults [111] in which poor sleep both acutely and over extended periods of time contributed to emotional dysregulation, decreased coordination, and reduced connectivity in the SAL and MOT networks.

This combination in which sleep quality has a larger impact than sleep quantity in terms of mean difference and number of affected networks could be explained by the importance of different stages of sleep which cannot be measured through accelerometery alone. Indeed, it is well known that deep and REM sleep plays an important role in synaptic plasticity [112]. Further, it may be that continuous sleep is important for the clearing of metabolic waste. Indeed, clearance is mediated by the glymphatic system [113] which appears to be more active during extended sleep periods [113, 114]. Considered in aggregate, the available evidence indicates that good sleep is associated with strong FC across multiple regions although it is very possible that this association bi-directional in nature.

### Strengths and Limitations

Strengths of this study center on our use of high-quality physiologic measurements in a large population of older adults. Indeed, maximal exercise tests to assess aerobic capacity, gold standard assessments of body composition, and objectively captured measures of physical activity and sleep are rarely found together in studies of this size. However, limitations also exist. Perhaps the most serious of these is the lack of harmonization of scanners across sites. Although we made efforts to reduce the impact of location by statistically controlling for location in both the physiological and connectivity variables, the observed site-based differences in resting state connectivity across all networks does potentially impact these results and could confound our findings. Additionally, the interconnected role of many of these lifestyle variables cannot be overstated. While statistically controlling to compare unstandardized residuals likely helped to establish the separate contribution of each variable for these analyses it is possible that the relationships are more complex than is “fixed” simply through statistical controls. Although not unique to this study, the cross-sectional nature of the study precludes drawing meaningful conclusions regarding the directionality of observed relations. Additionally, this exclusively older adult population had a relatively narrow range of physical activity levels (all were self-reported sedentary in the past year) and was free from many of the diseases and conditions that may have substantial effect on brain health in the larger population. Thus, our findings may not be representative of other groups, particularly younger and/or more active individuals. Further, we used a population level estimation equation which may over- or under-estimate individual capacity at a given workload. Moreover, utilizing “volitional” vs. “true” maximal capacity means that individuals may have quit the GXT assessment prior to their actual maximal capacity due to excessive perceived exertion or localized muscle fatigue. Additionally, the fact that the population comprised exclusively sedentary individuals may have limited our ability to observe differences in functional connectivity associated with greater daily activity, particularly higher intensity activity. The sleep-based data is further limited in that we only captured night-time sleep statistics and did not gather data on daytime napping. This may have contributed to an underestimation of total sleep time which could lead to mischaracterizing the relations between FC and sleep quantity and quality.

## Conclusion

In this sample of community dwelling older adults, greater cardiovascular fitness, but not greater total daily physical activity, was associated with stronger functional connectivity in brain regions believed to govern higher cognitive functions. Thus, the present findings reinforce the association between fitness and brain health. Also, as hypothesized, better sleep in terms of efficiency and number of wakeful periods per night was associated with stronger functional connectivity in key regions associated with brain health. Finally, total body fat percentage was surprisingly associated with higher connectivity in the DAN. These findings, in combination with similar findings reported by others, suggest that interventions to preserve brain health with increasing age should be focused on maintaining cardiovascular fitness and ensuring high quality sleep rather than simply increasing the total quantity of physical activity or controlling body composition. In particular, future research exploring longitudinal changes associated with interventions designed to improve fitness or sleep would be valuable to better understand causality of these associations. It would also be useful to evaluate the degree to which short term changes in behavior induce changes in functional connectivity.

## Supplementary Information

Additional file 1.

## Data Availability

Both the baseline data used here, and the longitudinal data from these participants, are held by the primary investigator at Washington University in St. Louis and can be acquired with a formal data request that includes a data sharing agreement across institutions/investigators and a formal proposed project outline.

## Abbreviations

AD: Alzheimer’s disease
BMI: Body mass index
BSDAN: Seitzman defined dorsal attentional network
BSDMN: Seitzman defined default mode network
CSF: Cerebral spinal fluid
DAN: Dorsal attentional network
DMN: Default mode network
DXA: Dual X-ray absorptiometry
ECN: Executive control network
FC: Functional connectivity
GXT: Graded exercise testing
MET: Metabolic equivalents of task
ml of O_2_/kg/min: Milliliters of oxygen per kilogram of body weight per minute
MOT: Motor control network
MRI: Magnetic resonance imaging
OSA: Obstructive sleep apnea
ROI: Region of interest
rs-fMRI: Resting state fMRI
SAL: Salience network
V02_max_: The amount of oxygen that the body can utilize during maximal effort
VAT: Visceral adipose tissue
VIS: Visual processing network
VM CPM: Vector magnitude counts per minute
WASO: Wake after sleep onset
