# Supplementary material for: Cardiorespiratory Fitness and Sleep, but not Physical Activity, are Associated with Functional Connectivity in Older Adults
Source: Sports Med Open. 2024 Oct 19;10:113. doi: 10.1186/s40798-024-00778-6 (PMC11490599; doi:10.1186/s40798-024-00778-6)
Supplement: Supplementary file 1 — Additional file 1. [file 40798_2024_778_MOESM1_ESM.docx]

SUPPLEMENTAL MATERIALS

Cardiorespiratory Fitness and Sleep, but not Physical Activity, are Associated with Functional Connectivity in Older Adults David Wing ^1,2^, Bart Roelands ^6,10,^ Julie Loebach Wetherell ^4,5^, Jeanne F Nichols^1,2^, Romain Meeusen ^6, 10,11^, Job G. Godino ^1,2^, Joshua S. Shimony ^9^, Abraham Z. Snyder ^9^, Tomoyuki Nishino ^3^, Ginger E Nicol ^3^, Guy Nagels ^8^, Lisa T. Eyler ^5,7^, and Eric J. Lenze ^3^

1 Herbert Wertheim School of Public Health and Human Longevity Science; University of California, San Diego

2 Exercise and Physical Activity Resource Center (EPARC); University of California, San Diego

3 Department of Psychiatry, Washington University School of Medicine, St. Louis, MO

4 Mental Health Service, VA San Diego Healthcare System

5 Department of Psychiatry, University of California, San Diego

6 Human Physiology & Sports Physiotherapy Research Group, Faculty of Physical Education and Physiotherapy, Vrije Universiteit Brussel, Brussels, Belgium

7 Desert-Pacific Mental Illness Research, Education, and Clinical Center, San Diego Veterans Administration Healthcare System, San Diego

8 Department of Neurology, UZ Brussel, Brussel, Belgium/Center for Neurosciences (C4N) Vrije Universiteit Brussel (VUB), Brussels, Belgium

9 Mallinckrodt Institute of Radiology, Washington University School of Medicine, St. Louis, MO

10: Brubotics, Vrije Universiteit Brussel, Brussels, Belgium

11: Department of Sports, Recreation, Exercise and Sciences, Community and Health Sciences; University of the Western Cape ,South Africa

Corresponding Author: David Wing. [dwing@eng.ucsd.edu](mailto:dwing@eng.ucsd.edu). 9500 Gilman Drive #0811. CALIT2/Atkinson Hall Room 3504. La Jolla, CA 92093-0811

Contents

[SUPPLEMENTARY TABLES 4](#_Toc175574295)

[Table 1: Difference in Variables of Interest by Site and Gender 4](#_Toc175574296)

[Tables 2 and 3: Correlations 5](#_Toc175574297)

[Behavioral/Physiological Variables of Interest 5](#_Toc175574298)

[Functional Networks 6](#_Toc175574299)

[Table 4: Talairach Locations and Designations to Networks Used 7](#_Toc175574300)

[SUPPLEMENTARY FIGURES: 7](#_Toc175574301)

[Seitzman and Voss Coordinates and ROI’s: Visual Representations 7](#_Toc175574302)

[Default Mode Network 7](#_Toc175574303)

[Executive Control Network 8](#_Toc175574304)

[Dorsal Attentional Network 9](#_Toc175574305)

[Salience Network 10](#_Toc175574306)

[Sensory Networks 11](#_Toc175574307)

[Supplementary Figure 6: Sample Size by Variable with Reason for Exclusion 12](#_Toc175574308)

[Supplementary Figures 7-15: Histograms of Population Distributions (Full Sample) 12](#_Toc175574309)

[Cardiorespiratory Fitness 12](#_Toc175574310)

[Percentage Body Fat 12](#_Toc175574311)

[Absolute Lean Tissue 13](#_Toc175574312)

[Absolute Visceral Adipose Tissue Mass 14](#_Toc175574313)

[Sleep Efficiency 14](#_Toc175574314)

[Total Sleep Time 15](#_Toc175574315)

[Wake After Sleep Onset_Total Minutes 15](#_Toc175574316)

[Wake After Sleep Onset_Number of Awakenings 16](#_Toc175574317)

[Total Movement Per Day 16](#_Toc175574318)

[Supplementary Figures 16-24: Histograms of Population Distributions (UCSD Only) 17](#_Toc175574319)

[Cardiorespiratory Fitness 17](#_Toc175574320)

[Percentage Body Fat 18](#_Toc175574321)

[Absolute Lean Tissue 18](#_Toc175574322)

[Absolute Visceral Adipose Tissue Mass 19](#_Toc175574323)

[Sleep Efficiency 20](#_Toc175574324)

[Total Sleep Time 20](#_Toc175574325)

[Wake After Sleep Onset_Total Minutes 21](#_Toc175574326)

[Wake After Sleep Onset_Number of Awakenings 22](#_Toc175574327)

[Total Movement Per Day 22](#_Toc175574328)

[Supplementary Figures 25-33: Histograms of Population Distributions (WUSTL Only) 23](#_Toc175574329)

[Cardiorespiratory Fitness 23](#_Toc175574330)

[Percentage Body Fat 24](#_Toc175574331)

[Absolute Lean Tissue 24](#_Toc175574332)

[Absolute Visceral Adipose Tissue Mass 25](#_Toc175574333)

[Sleep Efficiency 26](#_Toc175574334)

[Total Sleep Time 26](#_Toc175574335)

[Wake After Sleep Onset_Total Minutes 27](#_Toc175574336)

[Wake After Sleep Onset_Number of Awakenings 28](#_Toc175574337)

[Total Movement Per Day 28](#_Toc175574338)

[Supplementary Figures 34-41: Scatterplots of Physiological/Behavioral Variables with FC Networks 29](#_Toc175574339)

[Cardiorespiratory Fitness 29](#_Toc175574340)

[Percentage Body Fat 30](#_Toc175574341)

[Absolute Lean Tissue 30](#_Toc175574342)

[Absolute Visceral Adipose Tissue Mass 31](#_Toc175574343)

[Sleep Efficiency 31](#_Toc175574344)

[Total Sleep Time 32](#_Toc175574345)

[Wake After Sleep Onset_Total Minutes 32](#_Toc175574346)

[Wake After Sleep Onset_Number of Awakenings 33](#_Toc175574347)

[Non Significant Linear Regression of Unstandardized Residuals of behavioral variables and functional connectivity. 33](#_Toc175574348)

[CRF: METS 33](#_Toc175574349)

[Percent Body Fat 37](#_Toc175574350)

[Visceral Adipose Tissue 42](#_Toc175574351)

[Physical Activity (VM CPM) 46](#_Toc175574352)

[Sleep Efficiency 50](#_Toc175574353)

[Total Sleep Time 54](#_Toc175574354)

[Wake After Sleep Onset Time 58](#_Toc175574355)

[Wake After Sleep Onset Number 62](#_Toc175574356)

SUPPLEMENTARY TABLES

## Table 1: Difference in Variables of Interest by Site and Gender

| **Supplementary Table 1**: Mean Difference and Confidence Intervals for Significant Differences by Site and Gender | | | | |
| --- | --- | --- | --- | --- |
| SITE: UCSD-WUSTL | | | | |
|  | P | Mean Difference (CI) | 95% CI (Low) | 95% CI (high) |
| Maximal Cardiovascular Fitness (METS) | <.001 | -1.0 | -1.4 | -0.7 |
| DMN Connectivity | <.001 | 0.052 | 0.04 | 0.064 |
| ECN Connectivity | <.001 | 0.034 | 0.029 | 0.04 |
| DAN Connectivity | <.001 | 0.033 | 0.028 | 0.039 |
| SAL Connectivity | <.001 | 0.088 | 0.07 | 0.107 |
| MOT Connectivity | <.001 | 0.091 | 0.071 | 0.112 |
| VIS Connectivity | <.001 | 0.061 | 0.049 | 0.073 |
| BSDMN Connectivity | <.001 | 0.039 | 0.031 | 0.047 |
| BSDAN Connectivity | <.001 | 0.028 | 0.016 | 0.04 |
|  |  |  |  |  |
| Sex: Female-Male | | | | |
| Age (yrs) | 0.005 | -1.6 | -2.7 | -0.5 |
| Maximal Cardiovascular Fitness (METS) | <.001 | -1.4 | -1.8 | -0.9 |
| Body Fat (%) | <.001 | 9.4 | 7.9 | 10.9 |
| Lean Tissue (g) | <.001 | -15307.6 | -16643.2 | -13972 |
| Visceral Adipose Tissue (g) | <.001 | -1063 | -1240.4 | -885.6 |
| Total Movement (VM CPM) | 0.026 | 135.5 | 16.6 | 254.5 |
| DMN Connectivity | 0.019 | 0.019 | 0.003 | 0.034 |
| Yrs=Years; METS= Metabolic Equivalent of Task; VM= Vector Magnitude; CPM= Counts per minute; g=grams; min= minutes; DMN = Default Mode Network; ECN = Executive Control Network; DAN = Dorsal Attentional Network; SAL = Salience Network; MOT = Motor Control Network; VIS = Visual Network; BS = Ben Sietzman defined network. | | | | |

## Tables 2 and 3: Correlations

Table 2: Correlations between

Behavioral/Physiological Variables of Interest


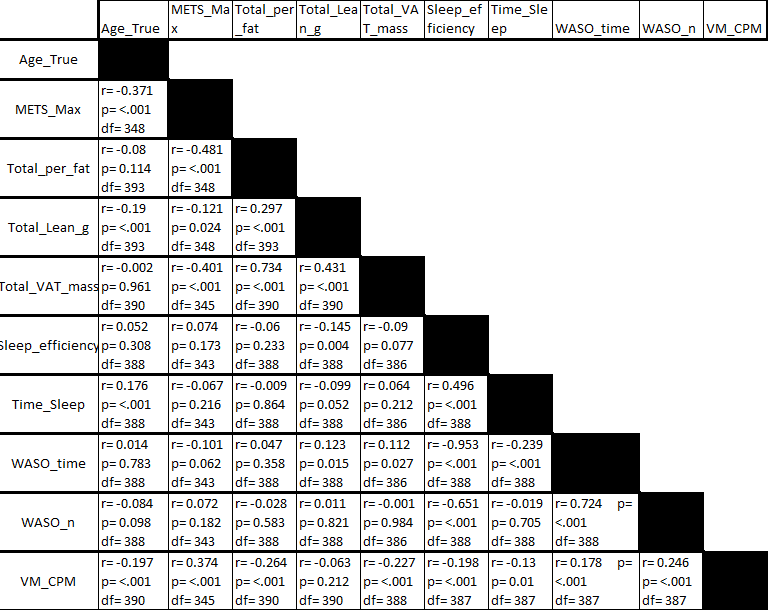


METS= Metabolic Equivalent of Task; VM= Vector Magnitude; CPM= Counts per minute; g=grams; WASO= Wake After Sleep Onset;

Table 3: Correlations between

Functional Networks


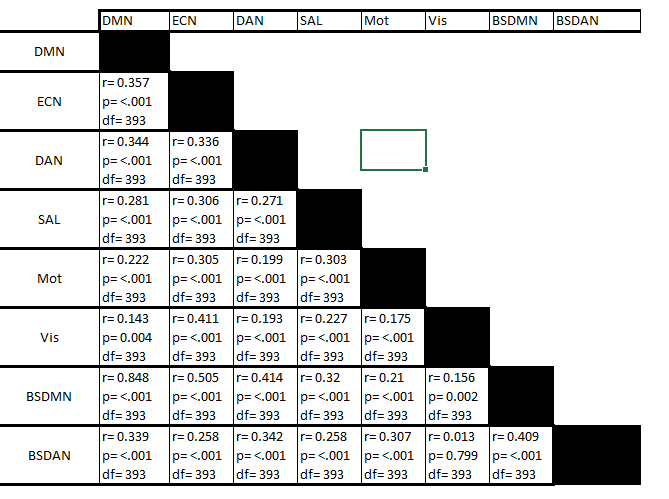


DMN = Default Mode Network; ECN = Executive Control Network; DAN = Dorsal Attentional Network; SAL = Salience Network; MOT = Motor Control Network; VIS = Visual Network; BS = Ben Sietzman defined network.

## Table 4: Talairach Locations and Designations to Networks Used

# SUPPLEMENTARY FIGURES:

## Seitzman and Voss Coordinates and ROI’s: Visual Representations

**Supplementary Figure 1**

Seitzman and Voss Coordinates and ROI’s

### Default Mode Network


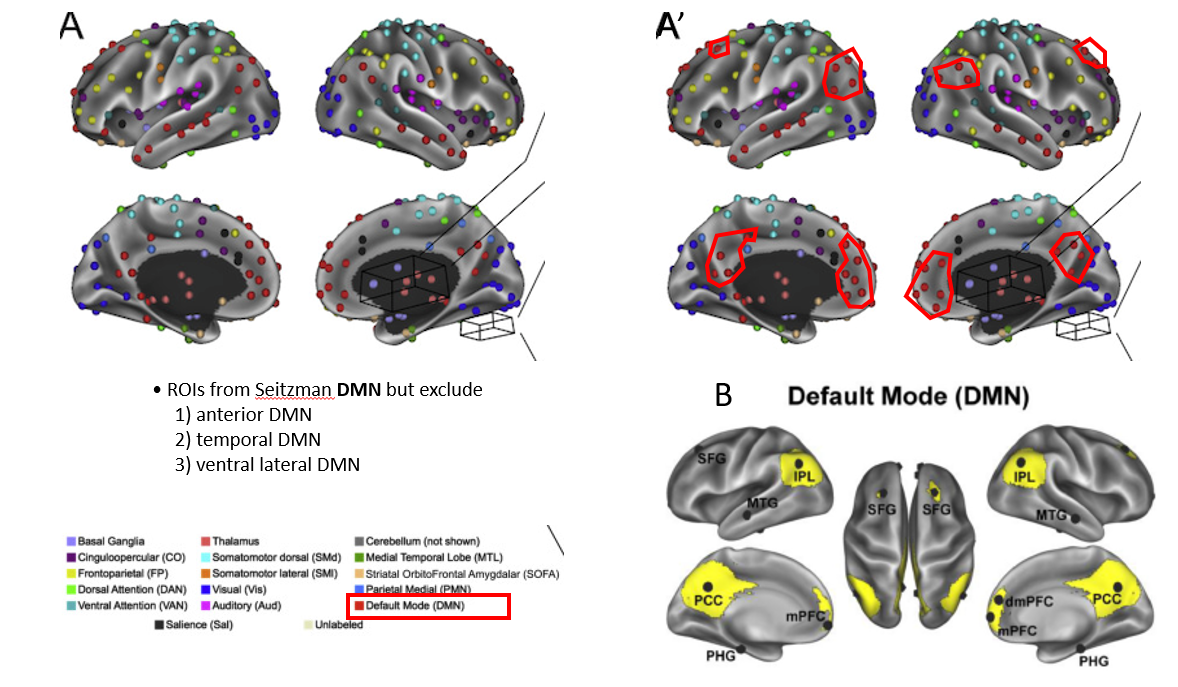
A=Seitzman coordinates and associated ROI designations

B=Visual representation of Voss ROI designations

A’=Overlay of Voss visual representations on Seitzman coordinates/designations

**Supplementary Figure 2**

Seitzman and Voss Coordinates and ROI’s

### Executive Control Network


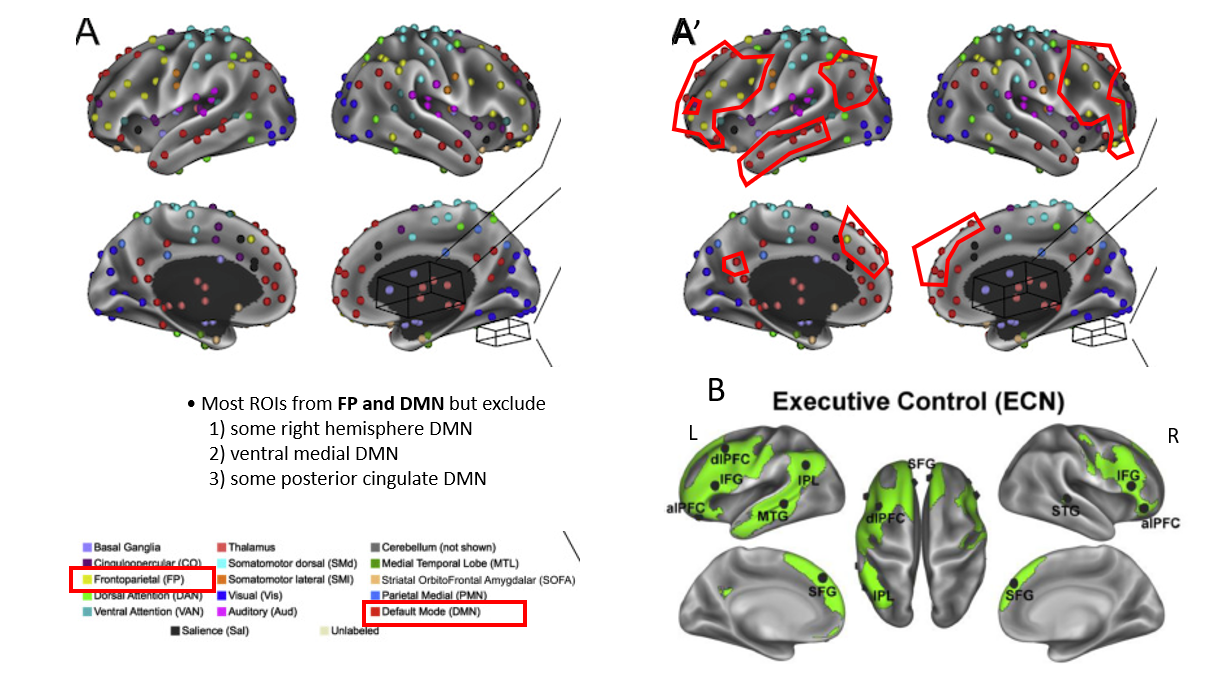
A=Seitzman coordinates and associated ROI designations

B=Visual representation of Voss ROI designations

A’=Overlay of Voss visual representations on Seitzman coordinates/designations

**Supplementary Figure 3**

Seitzman and Voss Coordinates and ROI’s

### Dorsal Attentional Network


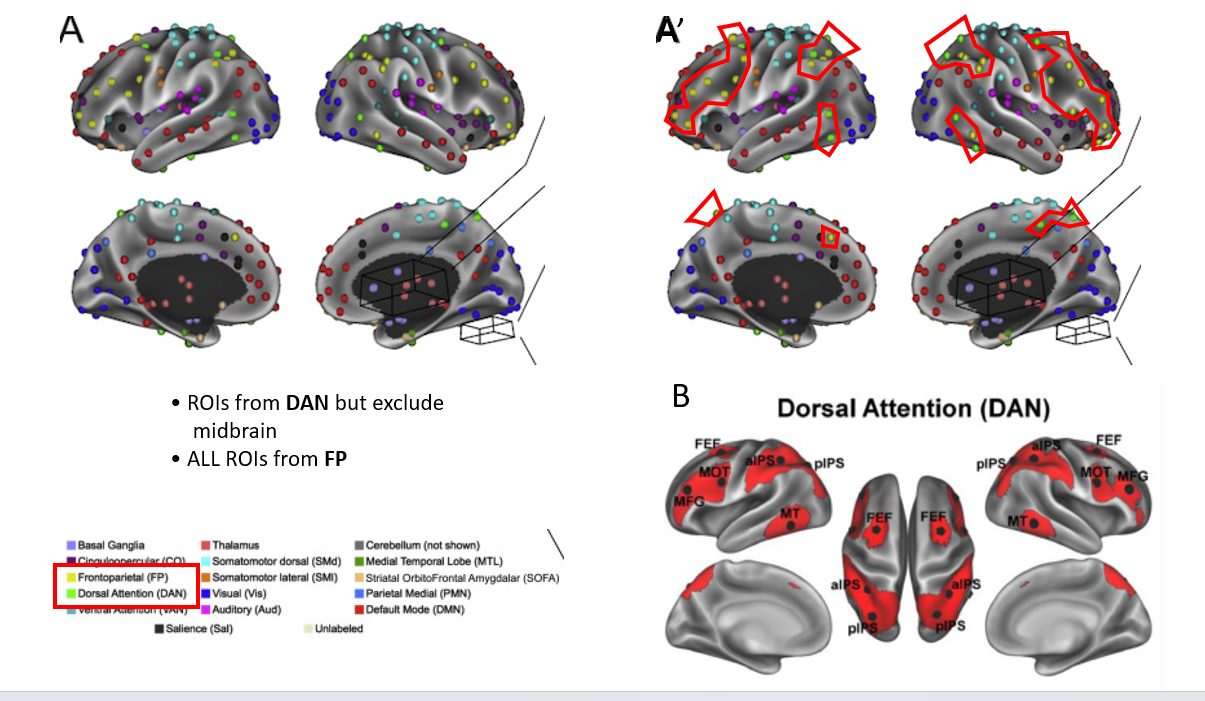


A=Seitzman coordinates and associated ROI designations

B=Visual representation of Voss ROI designations

A’=Overlay of Voss visual representations on Seitzman coordinates/designations

**Supplementary Figure 4**

Seitzman and Voss Coordinates and ROI’s

### Salience Network


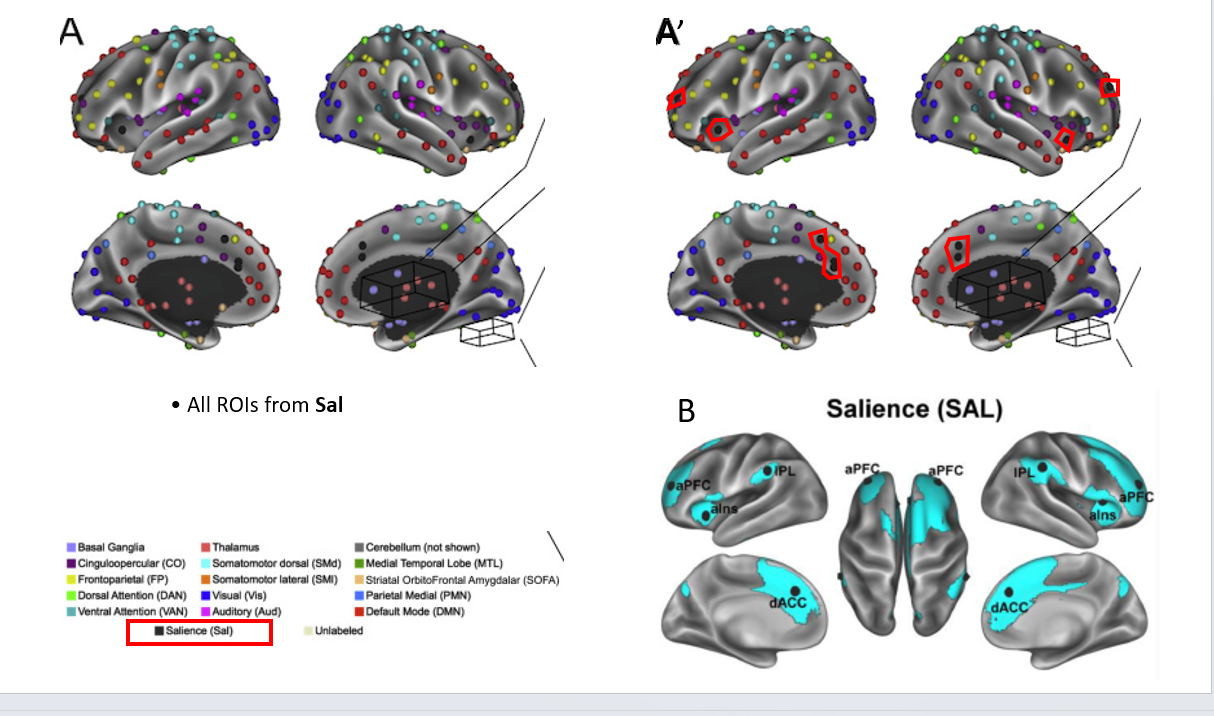


A=Seitzman coordinates and associated ROI designations

B=Visual representation of Voss ROI designations

A’=Overlay of Voss visual representations on Seitzman coordinates/designations

**Supplementary Figure 5**

Seitzman and Voss Coordinates and ROI’s

### Sensory Networks


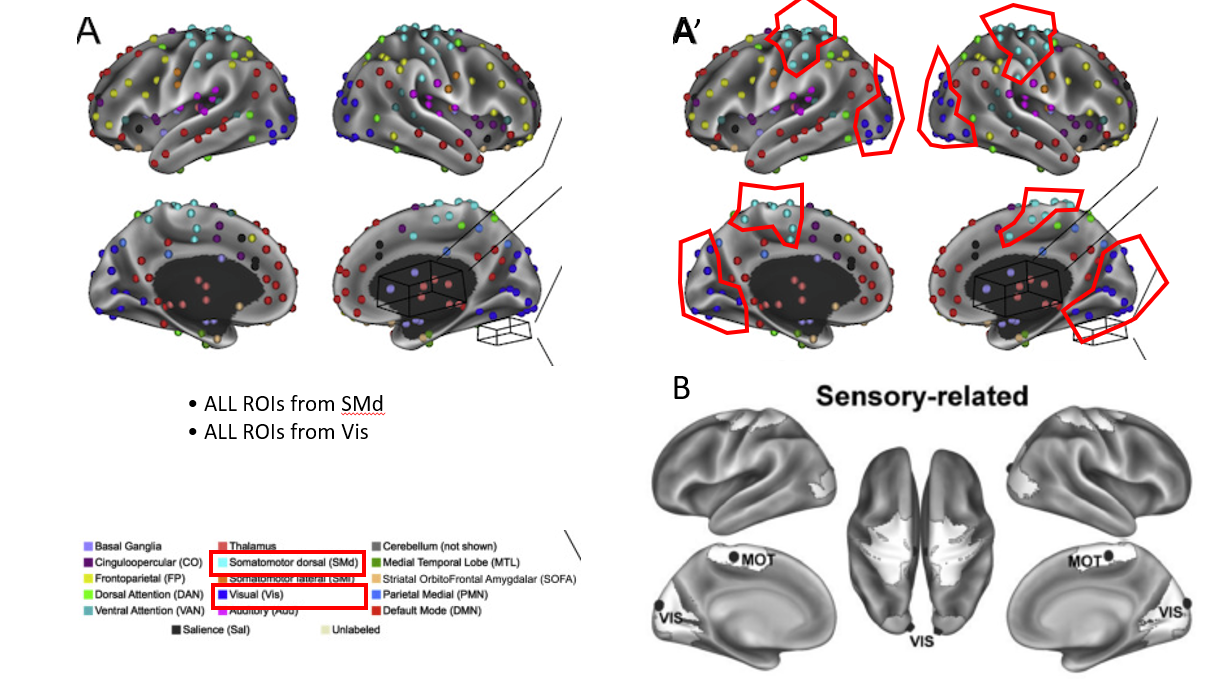


A=Seitzman coordinates and associated ROI designations

B=Visual representation of Voss ROI designations

A’=Overlay of Voss visual representations on Seitzman coordinates/designations

## Supplementary Figure 6: Sample Size by Variable with Reason for Exclusion


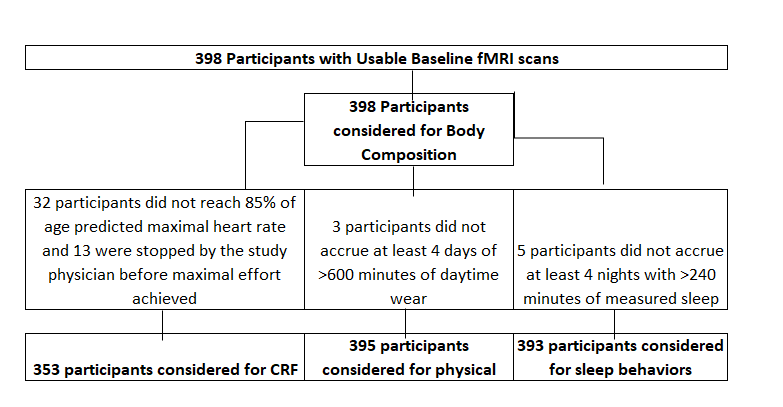


## Supplementary Figures 7-15: Histograms of Population Distributions (Full Sample)

### Cardiorespiratory Fitness


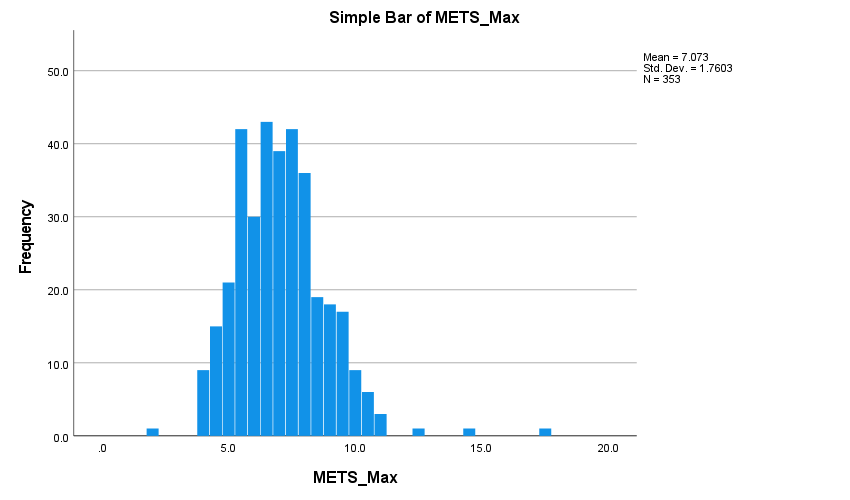


### Percentage Body Fat


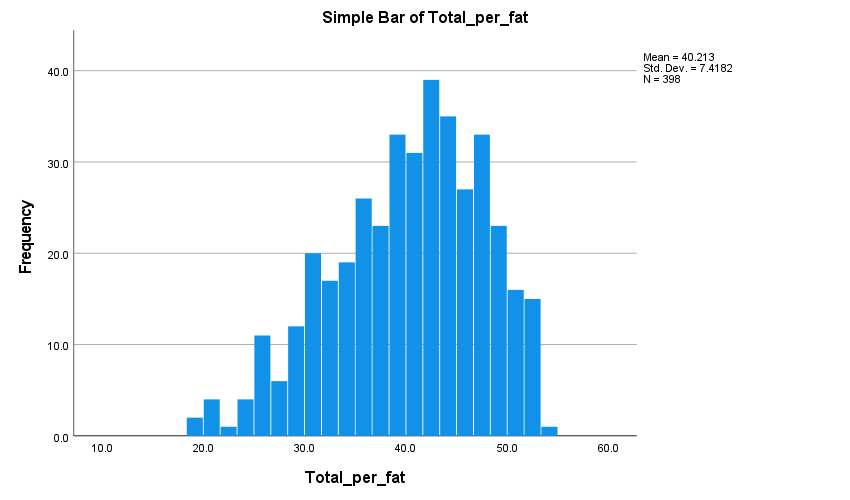


### Absolute Lean Tissue


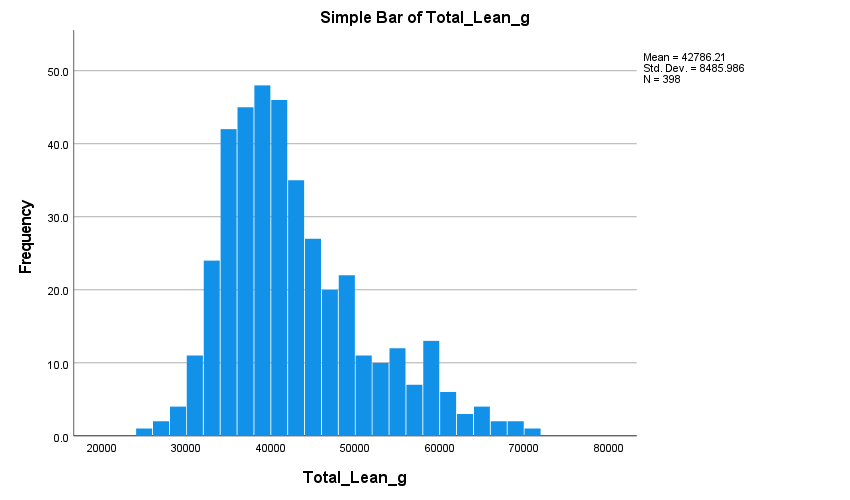


### Absolute Visceral Adipose Tissue Mass


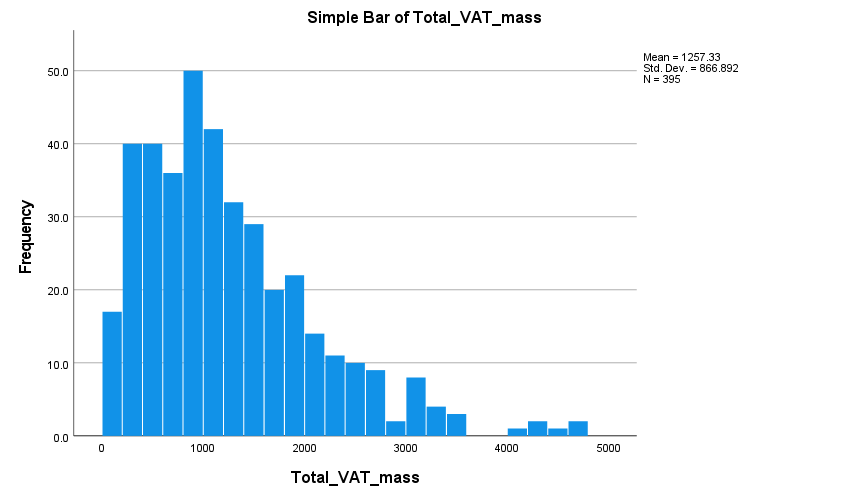


### Sleep Efficiency


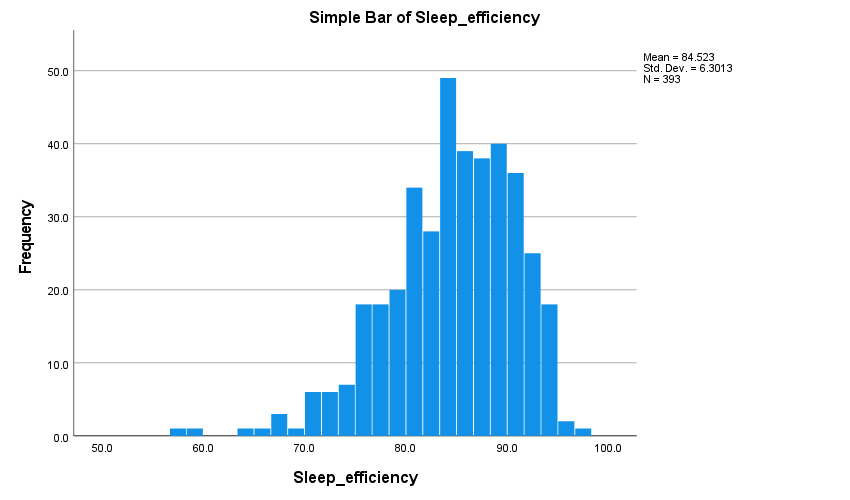


### Total Sleep Time


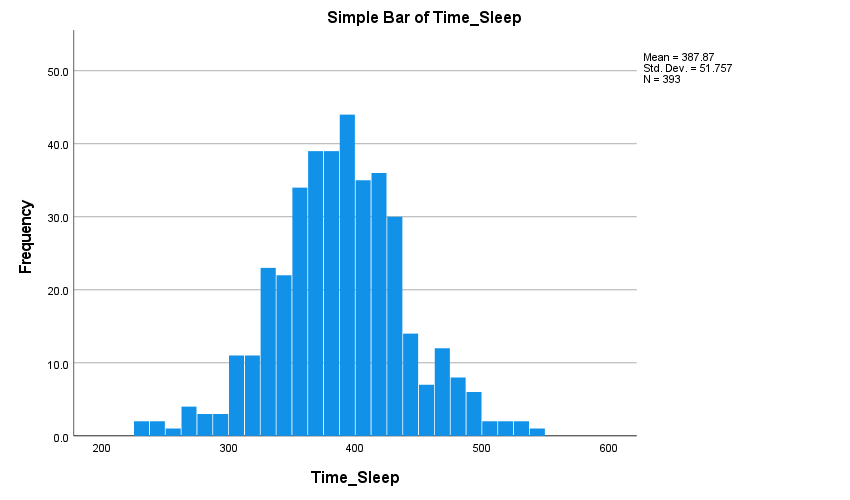


### Wake After Sleep Onset_Total Minutes


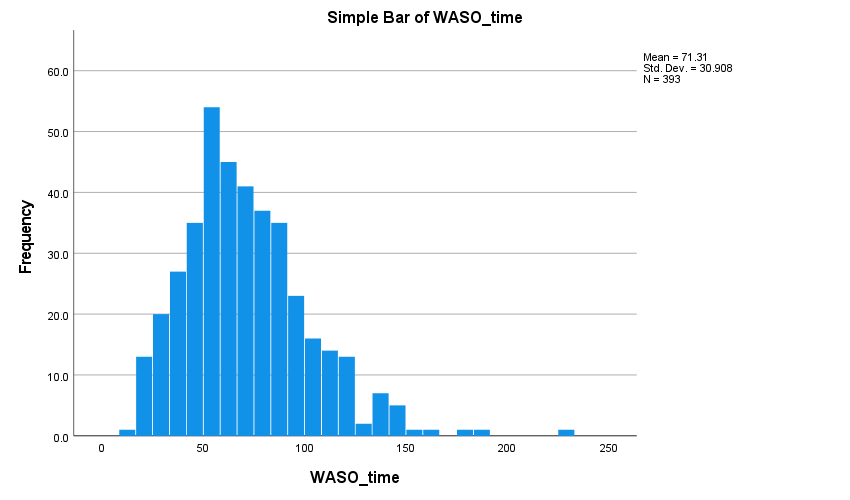


### Wake After Sleep Onset_Number of Awakenings


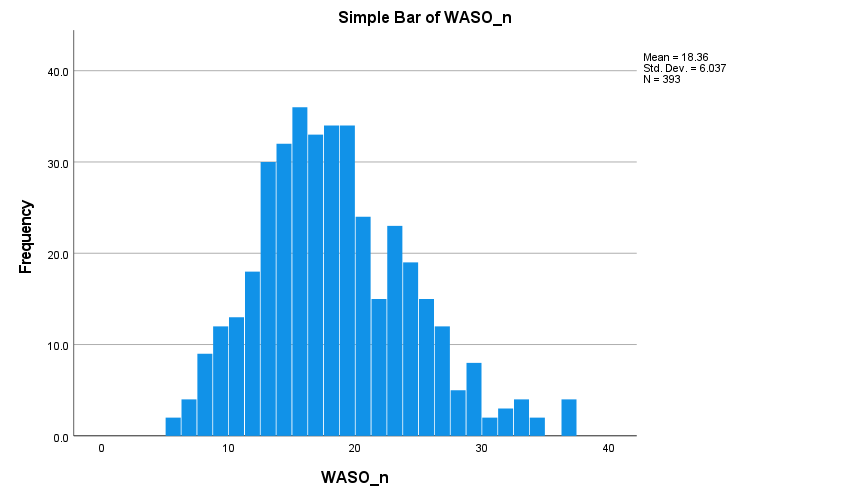


### Total Movement Per Day


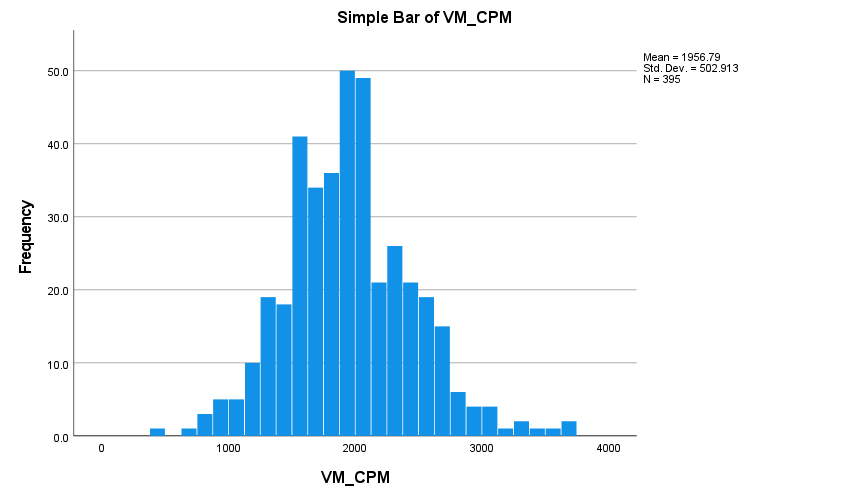


## Supplementary Figures 16-24: Histograms of Population Distributions (UCSD Only)

### Cardiorespiratory Fitness


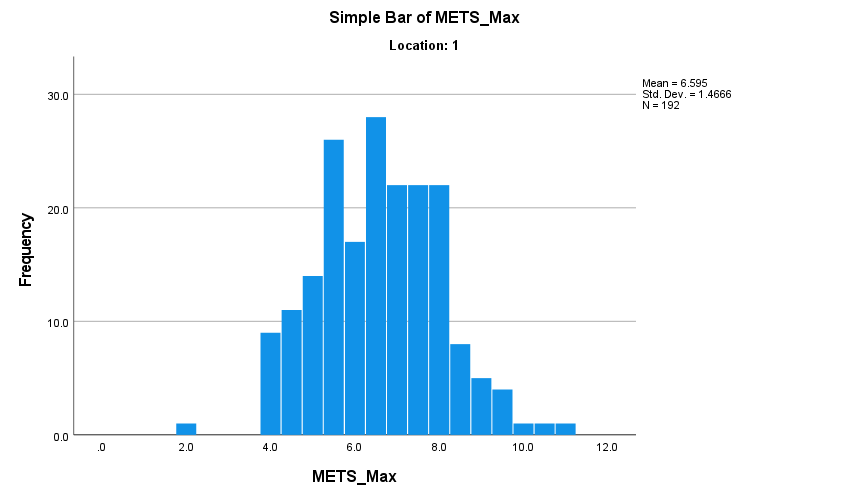


### Percentage Body Fat


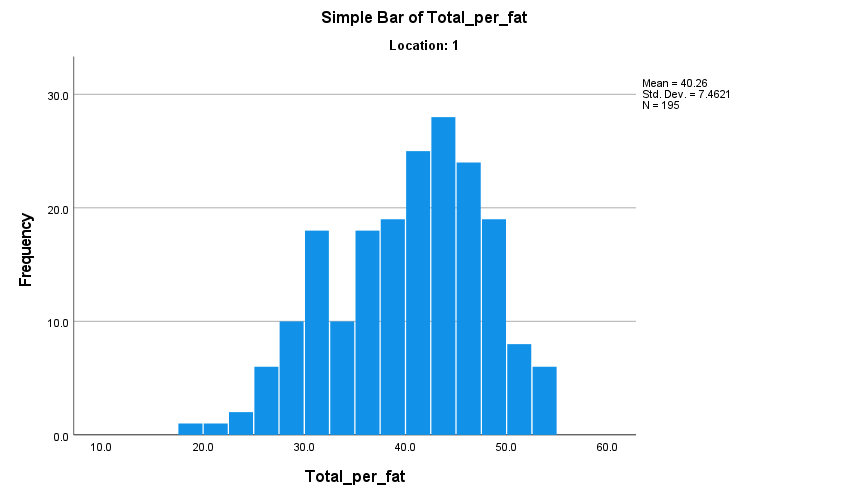


### Absolute Lean Tissue


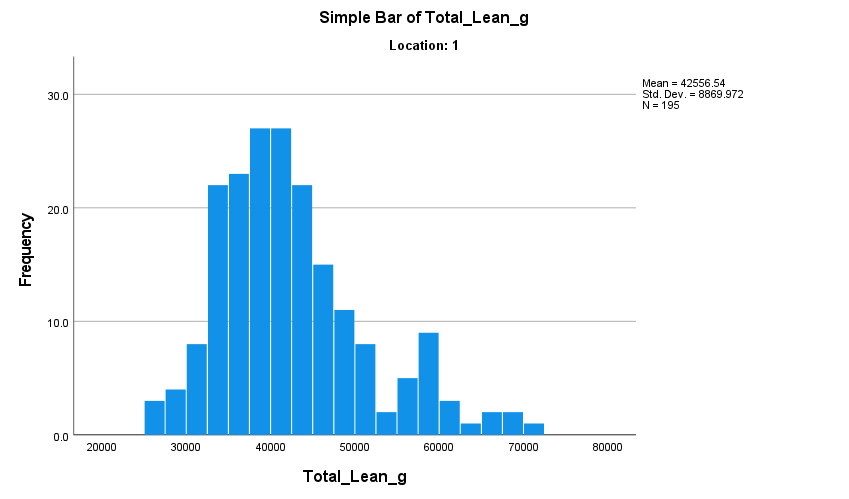


### Absolute Visceral Adipose Tissue Mass


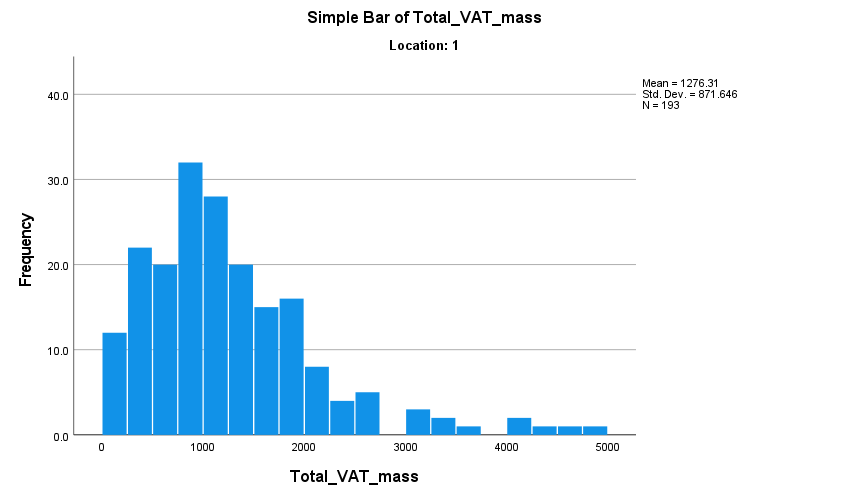


### Sleep Efficiency


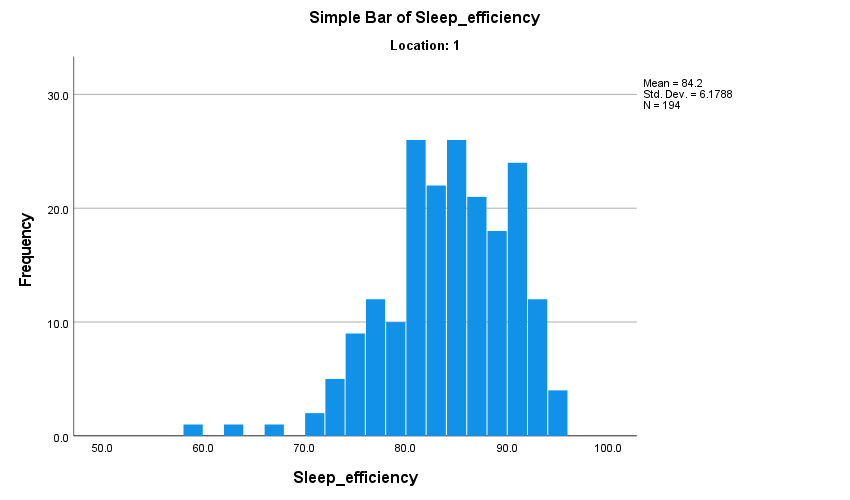


### Total Sleep Time


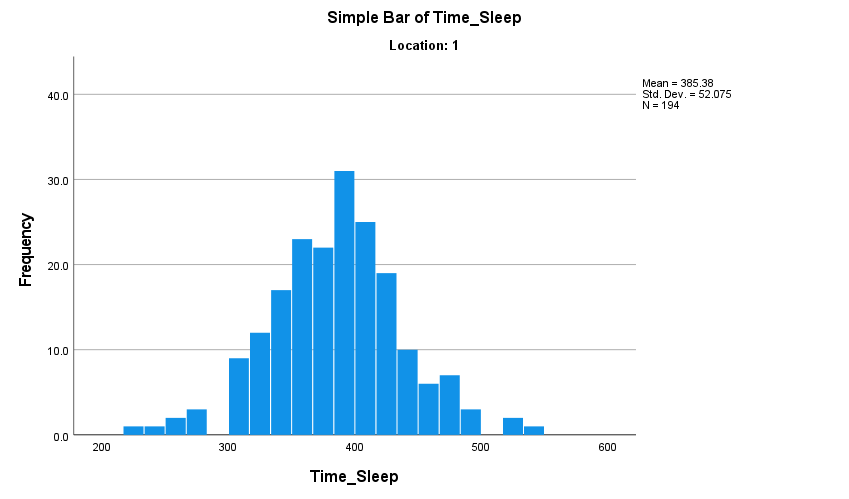


### Wake After Sleep Onset_Total Minutes


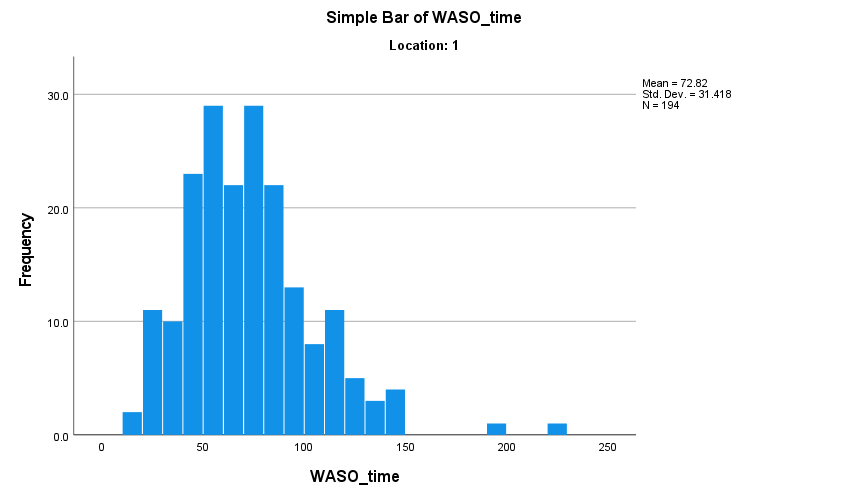


### Wake After Sleep Onset_Number of Awakenings


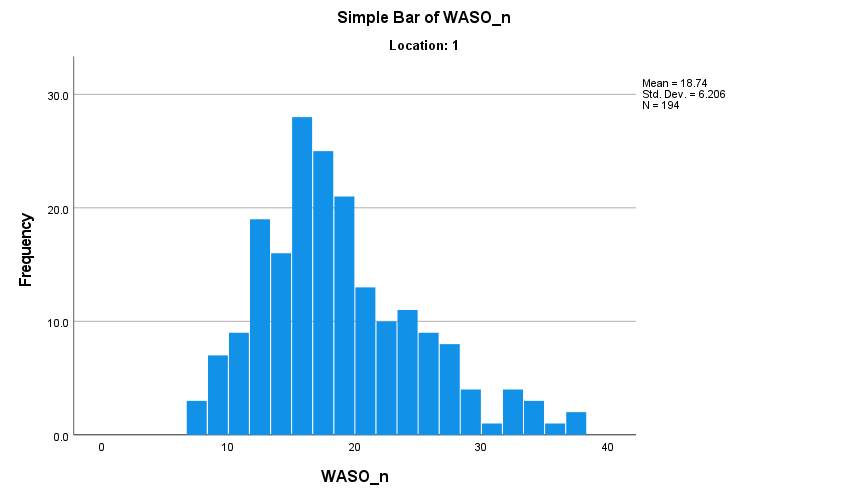


### Total Movement Per Day


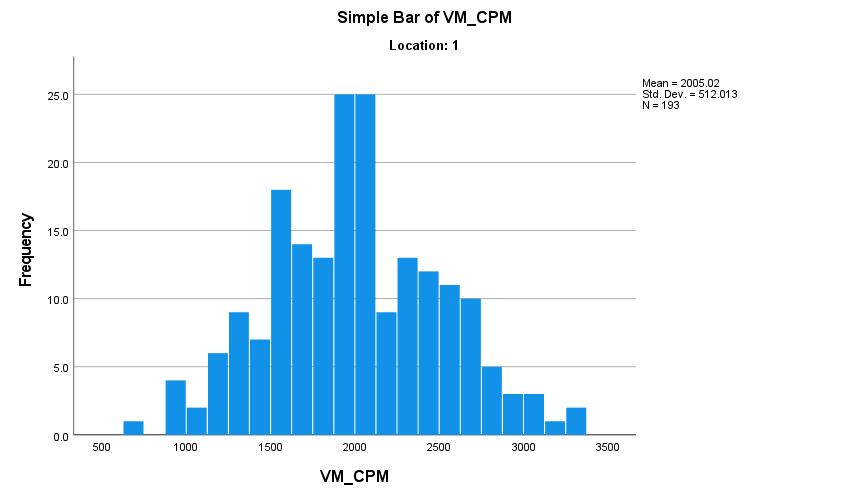


## Supplementary Figures 25-33: Histograms of Population Distributions (WUSTL Only)

### Cardiorespiratory Fitness


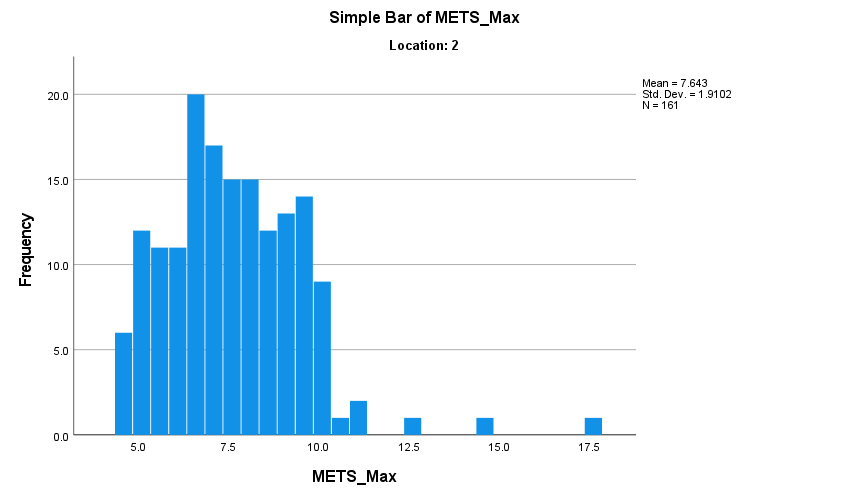


### Percentage Body Fat


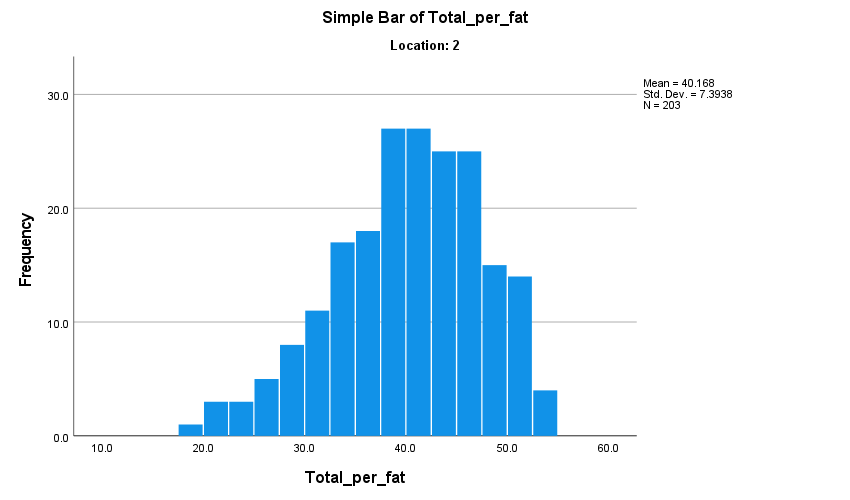


### Absolute Lean Tissue


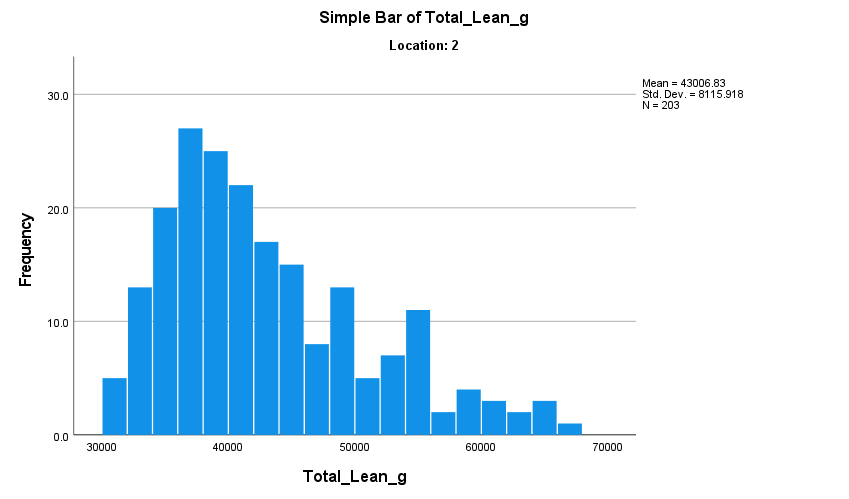


### Absolute Visceral Adipose Tissue Mass


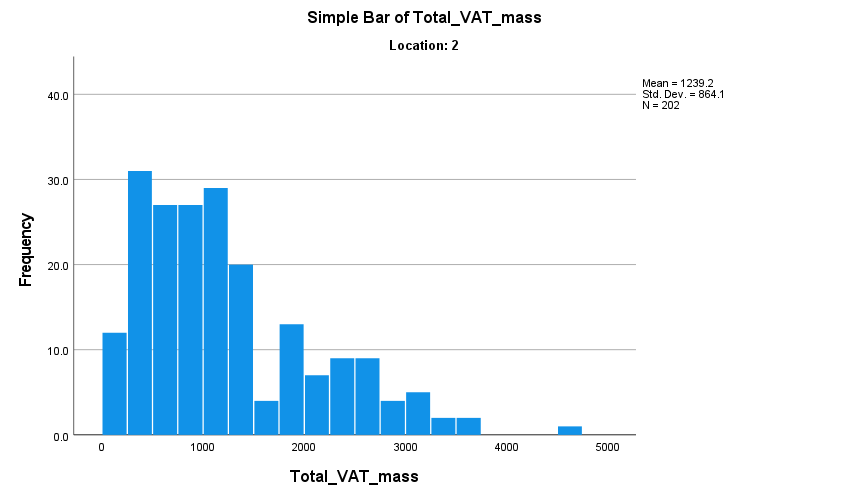


### Sleep Efficiency


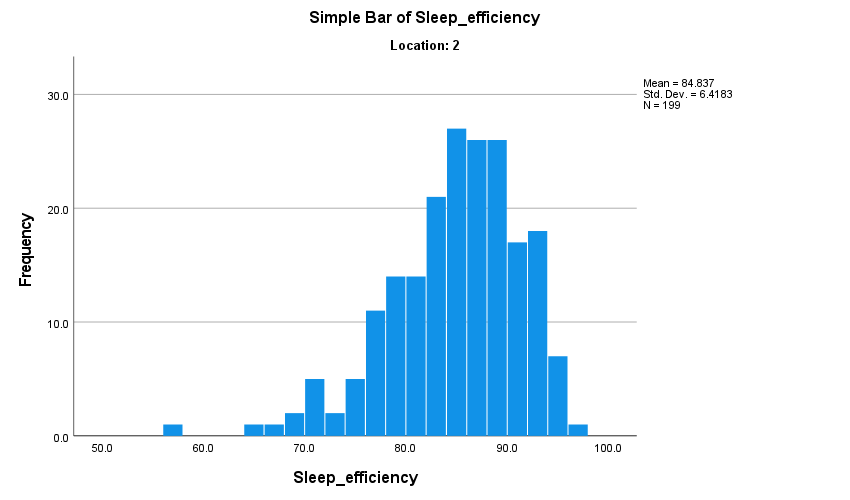


### Total Sleep Time


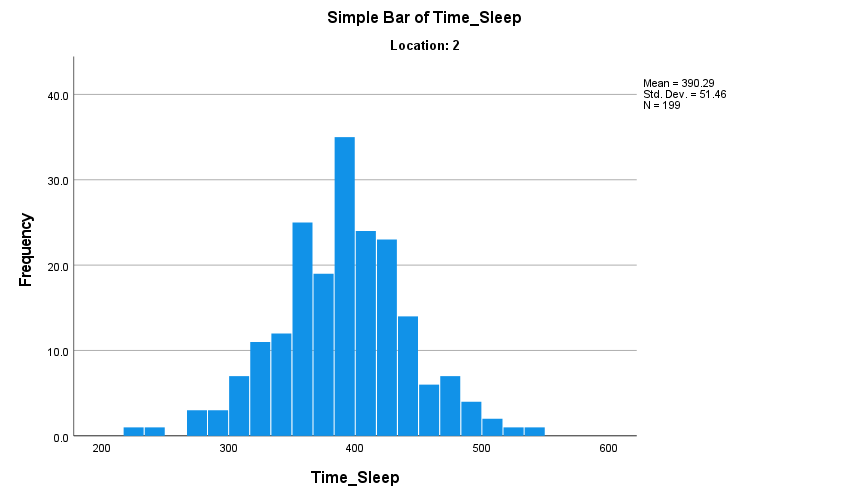


### Wake After Sleep Onset_Total Minutes


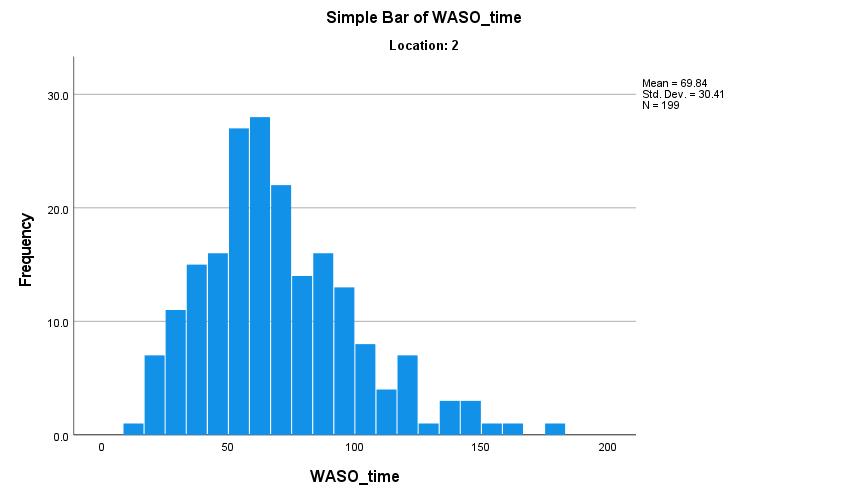


### Wake After Sleep Onset_Number of Awakenings


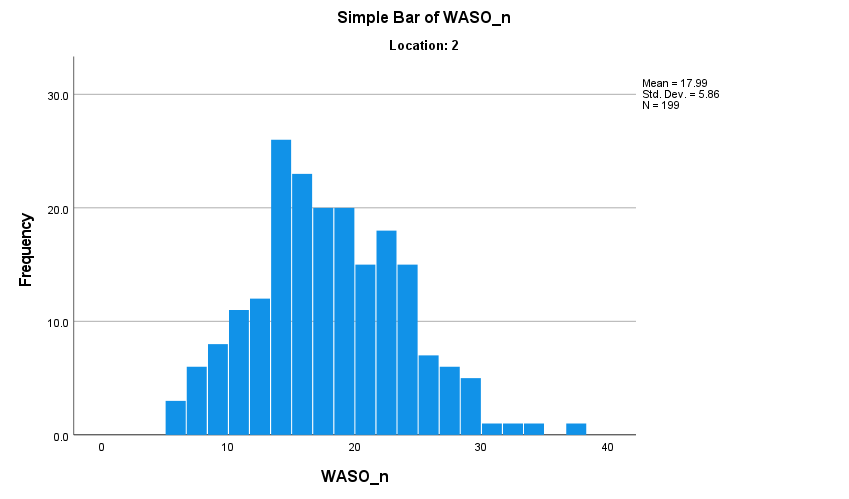


### Total Movement Per Day


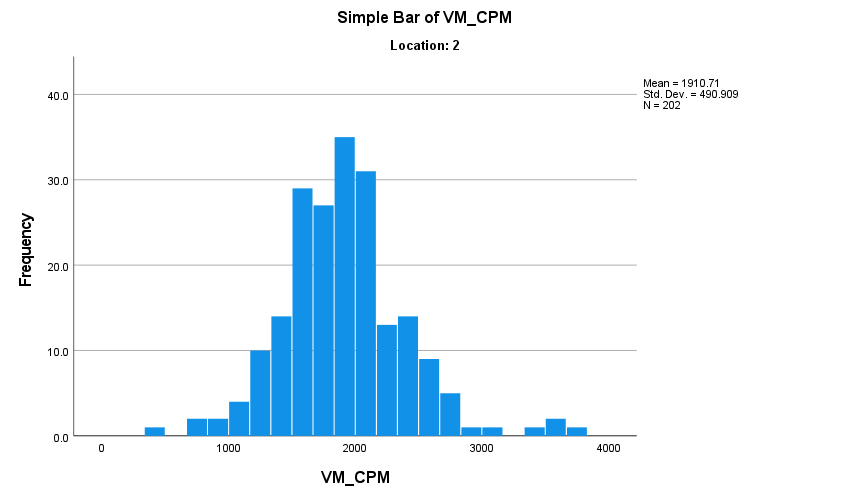


## Supplementary Figures 34-41: Scatterplots of Physiological/Behavioral Variables with FC Networks

### Cardiorespiratory Fitness


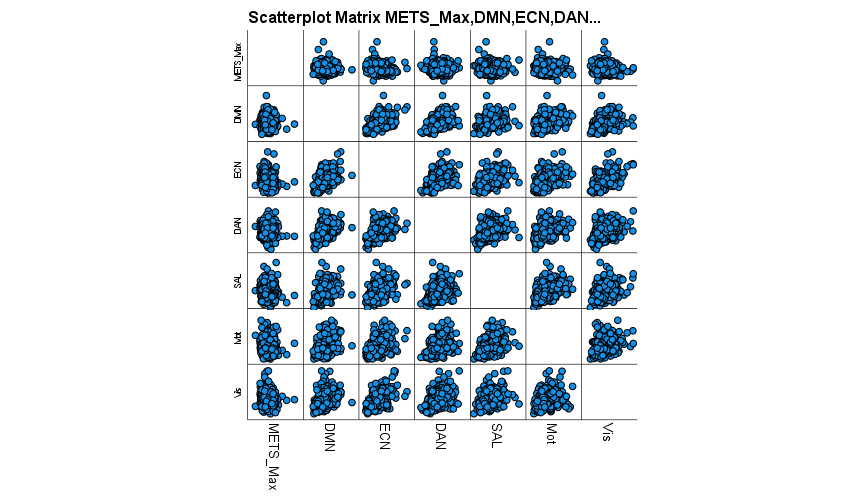


### Percentage Body Fat


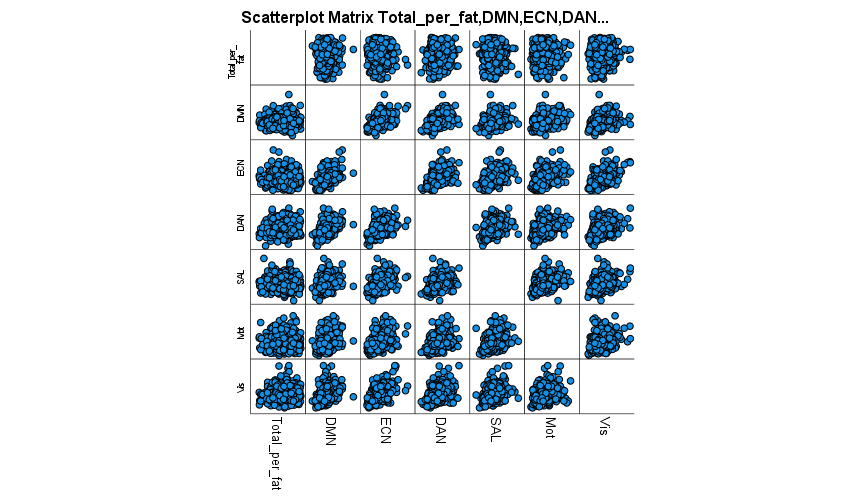


### Absolute Lean Tissue


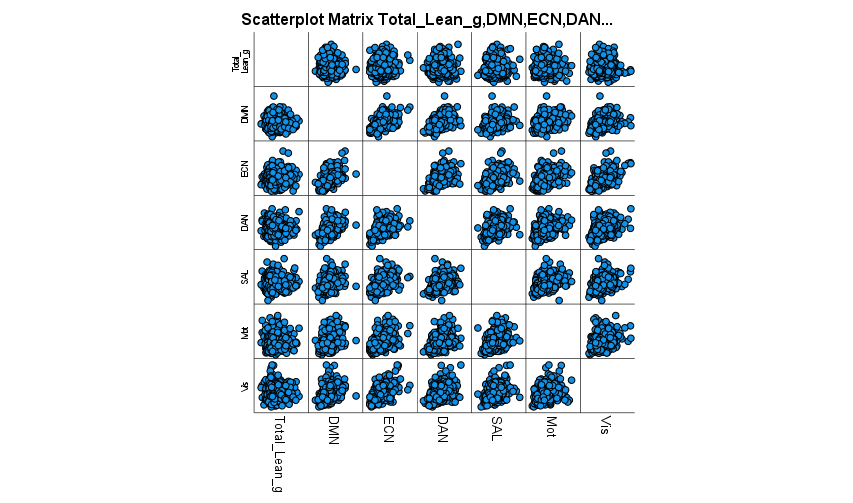


### Absolute Visceral Adipose Tissue Mass


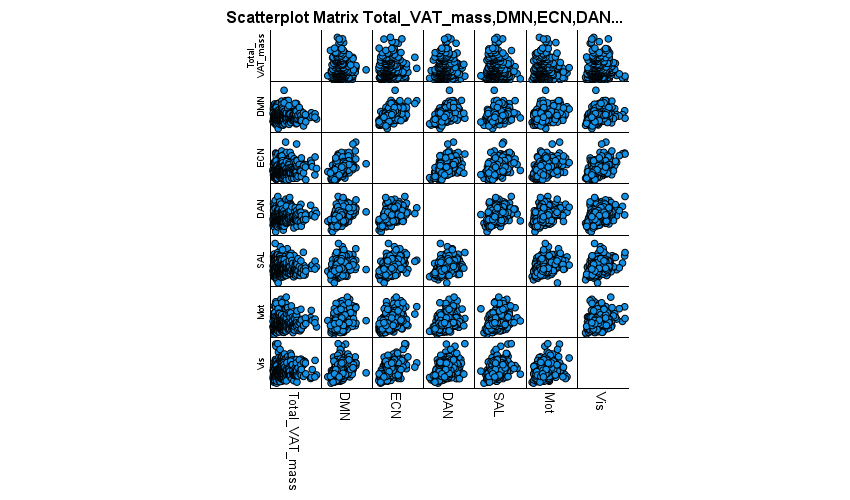


### Sleep Efficiency


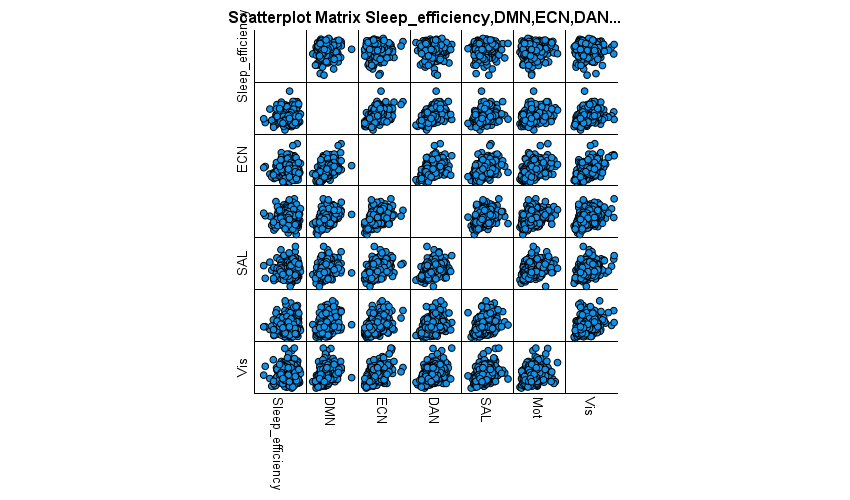


### Total Sleep Time


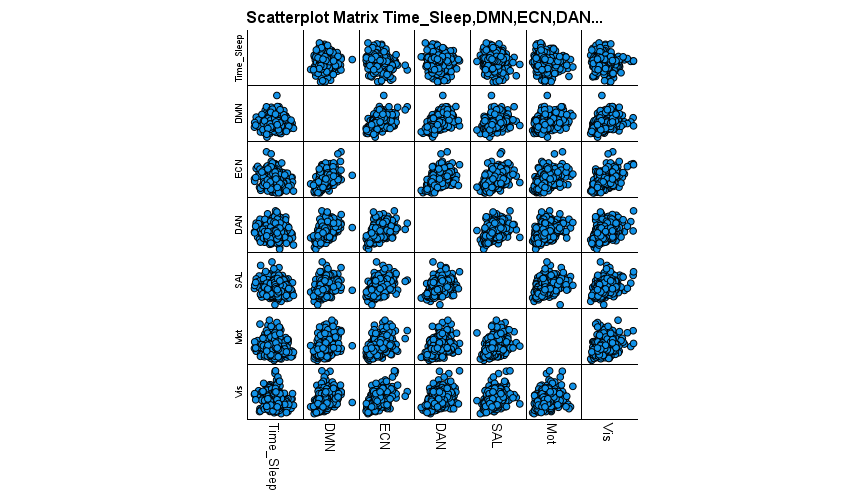


### Wake After Sleep Onset_Total Minutes


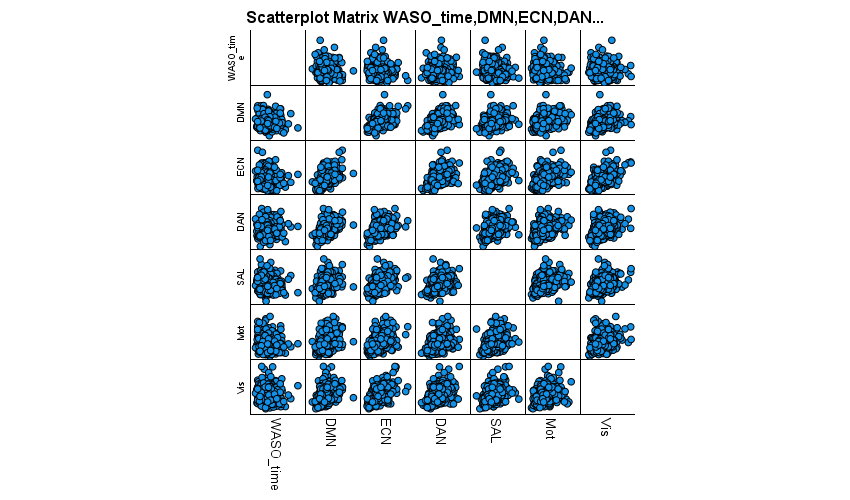


### Wake After Sleep Onset_Number of Awakenings


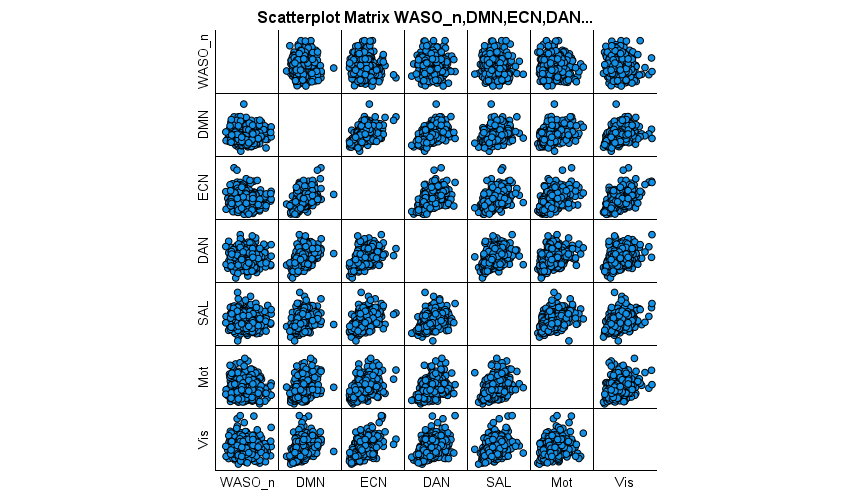


## Non Significant Linear Regression of Unstandardized Residuals of behavioral variables and functional connectivity.

NOTE: These will always be in the same order. Specifically: DMN, ECN, DAN, SAL, BSDMN, BSDAN, MOT, VIS

###

### CRF: METS

| **Model Summary^b^** | | | | |
| --- | --- | --- | --- | --- |
| Model | R | R Square | Adjusted R Square | Std. Error of the Estimate |
| 1 | .043^a^ | .002 | -.001 | .02796606 |
| a. Predictors: (Constant), New Mets Residual | | | | |
| b. Dependent Variable: ECN Residual | | | | |

| **Coefficients^a^** | | | | | | |
| --- | --- | --- | --- | --- | --- | --- |
| Model | | Unstandardized Coefficients | | Standardized Coefficients | t | Sig. |
|  |  | B | Std. Error | Beta |  |  |
| 1 | (Constant) | .001 | .002 |  | .333 | .739 |
|  | New Mets Residual | -.001 | .001 | -.043 | -.797 | .426 |
| a. Dependent Variable: ECN Residual | | | | | | |

| **Model Summary^b^** | | | | |
| --- | --- | --- | --- | --- |
| Model | R | R Square | Adjusted R Square | Std. Error of the Estimate |
| 1 | .013^a^ | .000 | -.003 | .02886753 |
| a. Predictors: (Constant), New Mets Residual | | | | |
| b. Dependent Variable: DAN Residual | | | | |

| **Coefficients^a^** | | | | | | |
| --- | --- | --- | --- | --- | --- | --- |
| Model | | Unstandardized Coefficients | | Standardized Coefficients | t | Sig. |
|  |  | B | Std. Error | Beta |  |  |
| 1 | (Constant) | .000 | .002 |  | -.108 | .914 |
|  | New Mets Residual | .000 | .001 | -.013 | -.236 | .814 |
| a. Dependent Variable: DAN Residual | | | | | | |

| **Model Summary^b^** | | | | |
| --- | --- | --- | --- | --- |
| Model | R | R Square | Adjusted R Square | Std. Error of the Estimate |
| 1 | .060^a^ | .004 | .001 | .08915250 |
| a. Predictors: (Constant), New Mets Residual | | | | |
| b. Dependent Variable: SAL Residual | | | | |

| **Coefficients^a^** | | | | | | |
| --- | --- | --- | --- | --- | --- | --- |
| Model | | Unstandardized Coefficients | | Standardized Coefficients | t | Sig. |
|  |  | B | Std. Error | Beta |  |  |
| 1 | (Constant) | .000 | .005 |  | -.045 | .964 |
|  | New Mets Residual | .004 | .003 | .060 | 1.114 | .266 |
| a. Dependent Variable: SAL Residual | | | | | | |

| **Model Summary^b^** | | | | |
| --- | --- | --- | --- | --- |
| Model | R | R Square | Adjusted R Square | Std. Error of the Estimate |
| 1 | .093^a^ | .009 | .006 | .04087723 |
| a. Predictors: (Constant), New Mets Residual | | | | |
| b. Dependent Variable: BSDMN Residual | | | | |

| **Coefficients^a^** | | | | | | |
| --- | --- | --- | --- | --- | --- | --- |
| Model | | Unstandardized Coefficients | | Standardized Coefficients | t | Sig. |
|  |  | B | Std. Error | Beta |  |  |
| 1 | (Constant) | .002 | .002 |  | .757 | .450 |
|  | New Mets Residual | .003 | .002 | .093 | 1.744 | .082 |
| a. Dependent Variable: BSDMN Residual | | | | | | |

| **Model Summary^b^** | | | | |
| --- | --- | --- | --- | --- |
| Model | R | R Square | Adjusted R Square | Std. Error of the Estimate |
| 1 | .022^a^ | .000 | -.002 | .06158743 |
| a. Predictors: (Constant), New Mets Residual | | | | |
| b. Dependent Variable: BSDAN Residual | | | | |

| **Coefficients^a^** | | | | | | |
| --- | --- | --- | --- | --- | --- | --- |
| Model | | Unstandardized Coefficients | | Standardized Coefficients | t | Sig. |
|  |  | B | Std. Error | Beta |  |  |
| 1 | (Constant) | .002 | .003 |  | .610 | .542 |
|  | New Mets Residual | -.001 | .002 | -.022 | -.412 | .680 |
| a. Dependent Variable: BSDAN Residual | | | | | | |

| **Model Summary^b^** | | | | |
| --- | --- | --- | --- | --- |
| Model | R | R Square | Adjusted R Square | Std. Error of the Estimate |
| 1 | .038^a^ | .001 | -.001 | .10289775 |
| a. Predictors: (Constant), New Mets Residual | | | | |
| b. Dependent Variable: MOT Residual | | | | |

| **Coefficients^a^** | | | | | | |
| --- | --- | --- | --- | --- | --- | --- |
| Model | | Unstandardized Coefficients | | Standardized Coefficients | t | Sig. |
|  |  | B | Std. Error | Beta |  |  |
| 1 | (Constant) | .001 | .006 |  | .095 | .924 |
|  | New Mets Residual | -.003 | .004 | -.038 | -.704 | .482 |
| a. Dependent Variable: MOT Residual | | | | | | |

| **Model Summary^b^** | | | | |
| --- | --- | --- | --- | --- |
| Model | R | R Square | Adjusted R Square | Std. Error of the Estimate |
| 1 | .028^a^ | .001 | -.002 | .06118516 |
| a. Predictors: (Constant), New Mets Residual | | | | |
| b. Dependent Variable: VIS Residual | | | | |

| **Coefficients^a^** | | | | | | |
| --- | --- | --- | --- | --- | --- | --- |
| Model | | Unstandardized Coefficients | | Standardized Coefficients | t | Sig. |
|  |  | B | Std. Error | Beta |  |  |
| 1 | (Constant) | -7.010E-5 | .003 |  | -.021 | .983 |
|  | New Mets Residual | .001 | .002 | .028 | .520 | .604 |
| a. Dependent Variable: VIS Residual | | | | | | |

### Percent Body Fat (Note: Salience Network Significant—included in main paper)

| **Model Summary** | | | | |
| --- | --- | --- | --- | --- |
| Model | R | R Square | Adjusted R Square | Std. Error of the Estimate |
| 1 | .003^a^ | .000 | -.003 | .05986550 |
| a. Predictors: (Constant), % Fat_Unstandardized Residual | | | | |

| **Coefficients^a^** | | | | | | |
| --- | --- | --- | --- | --- | --- | --- |
| Model | | Unstandardized Coefficients | | Standardized Coefficients | t | Sig. |
|  |  | B | Std. Error | Beta |  |  |
| 1 | (Constant) | 6.256E-17 | .003 |  | .000 | 1.000 |
|  | % Fat_Unstandardized Residual | -2.499E-5 | .000 | -.003 | -.052 | .958 |
| a. Dependent Variable: DMN Residual | | | | | | |

| **Model Summary** | | | | |
| --- | --- | --- | --- | --- |
| Model | R | R Square | Adjusted R Square | Std. Error of the Estimate |
| 1 | .079^a^ | .006 | .004 | .02738308 |
| a. Predictors: (Constant), % Fat_Unstandardized Residual | | | | |

| **Coefficients^a^** | | | | | | |
| --- | --- | --- | --- | --- | --- | --- |
| Model | | Unstandardized Coefficients | | Standardized Coefficients | t | Sig. |
|  |  | B | Std. Error | Beta |  |  |
| 1 | (Constant) | -8.593E-17 | .001 |  | .000 | 1.000 |
|  | % Fat_Unstandardized Residual | .000 | .000 | .079 | 1.583 | .114 |
| a. Dependent Variable: ECN Residual | | | | | | |

| **Model Summary** | | | | |
| --- | --- | --- | --- | --- |
| Model | R | R Square | Adjusted R Square | Std. Error of the Estimate |
| 1 | .094^a^ | .009 | .006 | .02846201 |
| a. Predictors: (Constant), % Fat_Unstandardized Residual | | | | |

| **Coefficients^a^** | | | | | | |
| --- | --- | --- | --- | --- | --- | --- |
| Model | | Unstandardized Coefficients | | Standardized Coefficients | t | Sig. |
|  |  | B | Std. Error | Beta |  |  |
| 1 | (Constant) | 3.637E-17 | .001 |  | .000 | 1.000 |
|  | % Fat_Unstandardized Residual | .000 | .000 | .094 | 1.880 | .061 |
| a. Dependent Variable: DAN Residual | | | | | | |

| **Model Summary** | | | | |
| --- | --- | --- | --- | --- |
| Model | R | R Square | Adjusted R Square | Std. Error of the Estimate |
| 1 | .049^a^ | .002 | .000 | .04061267 |
| a. Predictors: (Constant), % Fat_Unstandardized Residual | | | | |

| **Coefficients^a^** | | | | | | |
| --- | --- | --- | --- | --- | --- | --- |
| Model | | Unstandardized Coefficients | | Standardized Coefficients | t | Sig. |
|  |  | B | Std. Error | Beta |  |  |
| 1 | (Constant) | 1.459E-16 | .002 |  | .000 | 1.000 |
|  | % Fat_Unstandardized Residual | .000 | .000 | -.049 | -.982 | .326 |
| a. Dependent Variable: BSDMN Residual | | | | | | |

| **Model Summary** | | | | |
| --- | --- | --- | --- | --- |
| Model | R | R Square | Adjusted R Square | Std. Error of the Estimate |
| 1 | .001^a^ | .000 | -.003 | .06078732 |
| a. Predictors: (Constant), % Fat_Unstandardized Residual | | | | |

| **Coefficients^a^** | | | | | | |
| --- | --- | --- | --- | --- | --- | --- |
| Model | | Unstandardized Coefficients | | Standardized Coefficients | t | Sig. |
|  |  | B | Std. Error | Beta |  |  |
| 1 | (Constant) | -7.090E-17 | .003 |  | .000 | 1.000 |
|  | % Fat_Unstandardized Residual | -1.358E-5 | .000 | -.001 | -.028 | .978 |
| a. Dependent Variable: BSDAN Residual | | | | | | |

| **Model Summary** | | | | |
| --- | --- | --- | --- | --- |
| Model | R | R Square | Adjusted R Square | Std. Error of the Estimate |
| 1 | .020^a^ | .000 | -.002 | .10167867 |
| a. Predictors: (Constant), % Fat_Unstandardized Residual | | | | |

| **Coefficients^a^** | | | | | | |
| --- | --- | --- | --- | --- | --- | --- |
| Model | | Unstandardized Coefficients | | Standardized Coefficients | t | Sig. |
|  |  | B | Std. Error | Beta |  |  |
| 1 | (Constant) | 1.220E-16 | .005 |  | .000 | 1.000 |
|  | % Fat_Unstandardized Residual | .000 | .001 | .020 | .407 | .684 |
| a. Dependent Variable: MOT Residual | | | | | | |

| **Model Summary** | | | | |
| --- | --- | --- | --- | --- |
| Model | R | R Square | Adjusted R Square | Std. Error of the Estimate |
| 1 | .089^a^ | .008 | .005 | .06037617 |
| a. Predictors: (Constant), % Fat_Unstandardized Residual | | | | |

| **Coefficients^a^** | | | | | | |
| --- | --- | --- | --- | --- | --- | --- |
| Model | | Unstandardized Coefficients | | Standardized Coefficients | t | Sig. |
|  |  | B | Std. Error | Beta |  |  |
| 1 | (Constant) | 8.839E-17 | .003 |  | .000 | 1.000 |
|  | % Fat_Unstandardized Residual | .001 | .000 | .089 | 1.777 | .076 |
| a. Dependent Variable: VIS Residual | | | | | | |

### Visceral Adipose Tissue (Note: Salience Network significant and included in main paper)

| **Model Summary** | | | | |
| --- | --- | --- | --- | --- |
| Model | R | R Square | Adjusted R Square | Std. Error of the Estimate |
| 1 | .026^a^ | .001 | -.002 | .05979059 |
| a. Predictors: (Constant), Unstandardized Residual | | | | |

| **Coefficients^a^** | | | | | | |
| --- | --- | --- | --- | --- | --- | --- |
| Model | | Unstandardized Coefficients | | Standardized Coefficients | t | Sig. |
|  |  | B | Std. Error | Beta |  |  |
| 1 | (Constant) | .001 | .003 |  | .168 | .867 |
|  | Unstandardized Residual | 2.081E-6 | .000 | .026 | .514 | .607 |
| a. Dependent Variable: DMN Residual | | | | | | |

| **Model Summary** | | | | |
| --- | --- | --- | --- | --- |
| Model | R | R Square | Adjusted R Square | Std. Error of the Estimate |
| 1 | .070^a^ | .005 | .002 | .02741671 |
| a. Predictors: (Constant), Unstandardized Residual | | | | |

| **Coefficients^a^** | | | | | | |
| --- | --- | --- | --- | --- | --- | --- |
| Model | | Unstandardized Coefficients | | Standardized Coefficients | t | Sig. |
|  |  | B | Std. Error | Beta |  |  |
| 1 | (Constant) | .000 | .001 |  | .125 | .901 |
|  | Unstandardized Residual | 2.566E-6 | .000 | .070 | 1.383 | .167 |
| a. Dependent Variable: ECN Residual | | | | | | |

| **Model Summary** | | | | |
| --- | --- | --- | --- | --- |
| Model | R | R Square | Adjusted R Square | Std. Error of the Estimate |
| 1 | .080^a^ | .006 | .004 | .02859440 |
| a. Predictors: (Constant), Unstandardized Residual | | | | |

| **Coefficients^a^** | | | | | | |
| --- | --- | --- | --- | --- | --- | --- |
| Model | | Unstandardized Coefficients | | Standardized Coefficients | t | Sig. |
|  |  | B | Std. Error | Beta |  |  |
| 1 | (Constant) | -4.140E-5 | .001 |  | -.029 | .977 |
|  | Unstandardized Residual | 3.068E-6 | .000 | .080 | 1.586 | .114 |
| a. Dependent Variable: DAN Residual | | | | | | |

| **Model Summary** | | | | |
| --- | --- | --- | --- | --- |
| Model | R | R Square | Adjusted R Square | Std. Error of the Estimate |
| 1 | .111^a^ | .012 | .010 | .09113472 |
| a. Predictors: (Constant), Unstandardized Residual | | | | |

| **Coefficients^a^** | | | | | | |
| --- | --- | --- | --- | --- | --- | --- |
| Model | | Unstandardized Coefficients | | Standardized Coefficients | t | Sig. |
|  |  | B | Std. Error | Beta |  |  |
| 1 | (Constant) | .001 | .005 |  | .159 | .874 |
|  | Unstandardized Residual | -1.360E-5 | .000 | -.111 | -2.205 | .028 |
| a. Dependent Variable: SAL Residual | | | | | | |

| **Model Summary** | | | | |
| --- | --- | --- | --- | --- |
| Model | R | R Square | Adjusted R Square | Std. Error of the Estimate |
| 1 | .036^a^ | .001 | -.001 | .04056299 |
| a. Predictors: (Constant), Unstandardized Residual | | | | |

| **Coefficients^a^** | | | | | | |
| --- | --- | --- | --- | --- | --- | --- |
| Model | | Unstandardized Coefficients | | Standardized Coefficients | t | Sig. |
|  |  | B | Std. Error | Beta |  |  |
| 1 | (Constant) | .000 | .002 |  | .180 | .857 |
|  | Unstandardized Residual | -1.940E-6 | .000 | -.036 | -.707 | .480 |
| a. Dependent Variable: BSDMN Residual | | | | | | |

| **Model Summary** | | | | |
| --- | --- | --- | --- | --- |
| Model | R | R Square | Adjusted R Square | Std. Error of the Estimate |
| 1 | .026^a^ | .001 | -.002 | .06095895 |
| a. Predictors: (Constant), Unstandardized Residual | | | | |

| **Coefficients^a^** | | | | | | |
| --- | --- | --- | --- | --- | --- | --- |
| Model | | Unstandardized Coefficients | | Standardized Coefficients | t | Sig. |
|  |  | B | Std. Error | Beta |  |  |
| 1 | (Constant) | .000 | .003 |  | .034 | .973 |
|  | Unstandardized Residual | 2.107E-6 | .000 | .026 | .511 | .610 |
| a. Dependent Variable: BSDAN Residual | | | | | | |

| **Model Summary** | | | | |
| --- | --- | --- | --- | --- |
| Model | R | R Square | Adjusted R Square | Std. Error of the Estimate |
| 1 | .055^a^ | .003 | .000 | .10170571 |
| a. Predictors: (Constant), Unstandardized Residual | | | | |

| **Coefficients^a^** | | | | | | |
| --- | --- | --- | --- | --- | --- | --- |
| Model | | Unstandardized Coefficients | | Standardized Coefficients | t | Sig. |
|  |  | B | Std. Error | Beta |  |  |
| 1 | (Constant) | .000 | .005 |  | .055 | .956 |
|  | Unstandardized Residual | -7.460E-6 | .000 | -.055 | -1.084 | .279 |
| a. Dependent Variable: MOT Residual | | | | | | |

| **Model Summary** | | | | |
| --- | --- | --- | --- | --- |
| Model | R | R Square | Adjusted R Square | Std. Error of the Estimate |
| 1 | .058^a^ | .003 | .001 | .06064417 |
| a. Predictors: (Constant), Unstandardized Residual | | | | |

| **Coefficients^a^** | | | | | | |
| --- | --- | --- | --- | --- | --- | --- |
| Model | | Unstandardized Coefficients | | Standardized Coefficients | t | Sig. |
|  |  | B | Std. Error | Beta |  |  |
| 1 | (Constant) | .000 | .003 |  | .046 | .963 |
|  | Unstandardized Residual | 4.696E-6 | .000 | .058 | 1.144 | .253 |
| a. Dependent Variable: VIS Residual | | | | | | |

### Physical Activity (VM CPM) (Note: Motor network significantly predicted. Included in main paper)

| **Model Summary** | | | | |
| --- | --- | --- | --- | --- |
| Model | R | R Square | Adjusted R Square | Std. Error of the Estimate |
| 1 | .037^a^ | .001 | -.002 | .06083367 |
| a. Predictors: (Constant), New PA Residual | | | | |

| **Coefficients^a^** | | | | | | |
| --- | --- | --- | --- | --- | --- | --- |
| Model | | Unstandardized Coefficients | | Standardized Coefficients | t | Sig. |
|  |  | B | Std. Error | Beta |  |  |
| 1 | (Constant) | .002 | .003 |  | .675 | .500 |
|  | New PA Residual | 4.925E-6 | .000 | .037 | .680 | .497 |
| a. Dependent Variable: DMN Residual | | | | | | |

| **Model Summary** | | | | |
| --- | --- | --- | --- | --- |
| Model | R | R Square | Adjusted R Square | Std. Error of the Estimate |
| 1 | .059^a^ | .004 | .001 | .02794273 |
| a. Predictors: (Constant), New PA Residual | | | | |

| **Coefficients^a^** | | | | | | |
| --- | --- | --- | --- | --- | --- | --- |
| Model | | Unstandardized Coefficients | | Standardized Coefficients | t | Sig. |
|  |  | B | Std. Error | Beta |  |  |
| 1 | (Constant) | .001 | .002 |  | .334 | .739 |
|  | New PA Residual | -3.664E-6 | .000 | -.059 | -1.101 | .272 |
| a. Dependent Variable: ECN Residual | | | | | | |

| **Model Summary** | | | | |
| --- | --- | --- | --- | --- |
| Model | R | R Square | Adjusted R Square | Std. Error of the Estimate |
| 1 | .058^a^ | .003 | .000 | .02882188 |
| a. Predictors: (Constant), New PA Residual | | | | |

| **Coefficients^a^** | | | | | | |
| --- | --- | --- | --- | --- | --- | --- |
| Model | | Unstandardized Coefficients | | Standardized Coefficients | t | Sig. |
|  |  | B | Std. Error | Beta |  |  |
| 1 | (Constant) | .000 | .002 |  | -.108 | .914 |
|  | New PA Residual | 3.680E-6 | .000 | .058 | 1.072 | .284 |
| a. Dependent Variable: DAN Residual | | | | | | |

| **Model Summary** | | | | |
| --- | --- | --- | --- | --- |
| Model | R | R Square | Adjusted R Square | Std. Error of the Estimate |
| 1 | .038^a^ | .001 | -.001 | .08924673 |
| a. Predictors: (Constant), New PA Residual | | | | |

| **Coefficients^a^** | | | | | | |
| --- | --- | --- | --- | --- | --- | --- |
| Model | | Unstandardized Coefficients | | Standardized Coefficients | t | Sig. |
|  |  | B | Std. Error | Beta |  |  |
| 1 | (Constant) | .000 | .005 |  | -.045 | .964 |
|  | New PA Residual | 7.590E-6 | .000 | .038 | .714 | .476 |
| a. Dependent Variable: SAL Residual | | | | | | |

| **Model Summary** | | | | |
| --- | --- | --- | --- | --- |
| Model | R | R Square | Adjusted R Square | Std. Error of the Estimate |
| 1 | .035^a^ | .001 | -.002 | .04103218 |
| a. Predictors: (Constant), New PA Residual | | | | |

| **Coefficients^a^** | | | | | | |
| --- | --- | --- | --- | --- | --- | --- |
| Model | | Unstandardized Coefficients | | Standardized Coefficients | t | Sig. |
|  |  | B | Std. Error | Beta |  |  |
| 1 | (Constant) | .002 | .002 |  | .754 | .451 |
|  | New PA Residual | 3.155E-6 | .000 | .035 | .646 | .519 |
| a. Dependent Variable: BSDMN Residual | | | | | | |

| **Model Summary** | | | | |
| --- | --- | --- | --- | --- |
| Model | R | R Square | Adjusted R Square | Std. Error of the Estimate |
| 1 | .073^a^ | .005 | .002 | .06143886 |
| a. Predictors: (Constant), New PA Residual | | | | |

| **Coefficients^a^** | | | | | | |
| --- | --- | --- | --- | --- | --- | --- |
| Model | | Unstandardized Coefficients | | Standardized Coefficients | t | Sig. |
|  |  | B | Std. Error | Beta |  |  |
| 1 | (Constant) | .002 | .003 |  | .612 | .541 |
|  | New PA Residual | 9.928E-6 | .000 | .073 | 1.357 | .176 |
| a. Dependent Variable: BSDAN Residual | | | | | | |

| **Model Summary** | | | | |
| --- | --- | --- | --- | --- |
| Model | R | R Square | Adjusted R Square | Std. Error of the Estimate |
| 1 | .063^a^ | .004 | .001 | .06108888 |
| a. Predictors: (Constant), New PA Residual | | | | |

| **Coefficients^a^** | | | | | | |
| --- | --- | --- | --- | --- | --- | --- |
| Model | | Unstandardized Coefficients | | Standardized Coefficients | t | Sig. |
|  |  | B | Std. Error | Beta |  |  |
| 1 | (Constant) | -7.010E-5 | .003 |  | -.021 | .983 |
|  | New PA Residual | -8.481E-6 | .000 | -.063 | -1.166 | .244 |
| a. Dependent Variable: VIS Residual | | | | | | |

### Sleep Efficiency (Note: Both Salience and Motor Network Significantly Predicted and included in main paper)

| **Model Summary** | | | | |
| --- | --- | --- | --- | --- |
| Model | R | R Square | Adjusted R Square | Std. Error of the Estimate |
| 1 | .048^a^ | .002 | -.001 | .06080337 |
| a. Predictors: (Constant), New Sleep % Residual | | | | |

| **Coefficients^a^** | | | | | | |
| --- | --- | --- | --- | --- | --- | --- |
| Model | | Unstandardized Coefficients | | Standardized Coefficients | t | Sig. |
|  |  | B | Std. Error | Beta |  |  |
| 1 | (Constant) | .002 | .003 |  | .676 | .500 |
|  | New Sleep % Residual | .000 | .001 | .048 | .898 | .370 |
| a. Dependent Variable: DMN Residual | | | | | | |

| **Model Summary** | | | | |
| --- | --- | --- | --- | --- |
| Model | R | R Square | Adjusted R Square | Std. Error of the Estimate |
| 1 | .030^a^ | .001 | -.002 | .02797936 |
| a. Predictors: (Constant), New Sleep % Residual | | | | |

| **Coefficients^a^** | | | | | | |
| --- | --- | --- | --- | --- | --- | --- |
| Model | | Unstandardized Coefficients | | Standardized Coefficients | t | Sig. |
|  |  | B | Std. Error | Beta |  |  |
| 1 | (Constant) | .001 | .002 |  | .333 | .739 |
|  | New Sleep % Residual | .000 | .000 | .030 | .554 | .580 |
| a. Dependent Variable: ECN Residual | | | | | | |

| **Model Summary** | | | | |
| --- | --- | --- | --- | --- |
| Model | R | R Square | Adjusted R Square | Std. Error of the Estimate |
| 1 | .052^a^ | .003 | .000 | .02883143 |
| a. Predictors: (Constant), New Sleep % Residual | | | | |

| **Coefficients^a^** | | | | | | |
| --- | --- | --- | --- | --- | --- | --- |
| Model | | Unstandardized Coefficients | | Standardized Coefficients | t | Sig. |
|  |  | B | Std. Error | Beta |  |  |
| 1 | (Constant) | .000 | .002 |  | -.108 | .914 |
|  | New Sleep % Residual | .000 | .000 | .052 | .959 | .338 |
| a. Dependent Variable: DAN Residual | | | | | | |

| **Model Summary** | | | | |
| --- | --- | --- | --- | --- |
| Model | R | R Square | Adjusted R Square | Std. Error of the Estimate |
| 1 | .103^a^ | .011 | .008 | .04083887 |
| a. Predictors: (Constant), New Sleep % Residual | | | | |

| **Coefficients^a^** | | | | | | |
| --- | --- | --- | --- | --- | --- | --- |
| Model | | Unstandardized Coefficients | | Standardized Coefficients | t | Sig. |
|  |  | B | Std. Error | Beta |  |  |
| 1 | (Constant) | .002 | .002 |  | .757 | .449 |
|  | New Sleep % Residual | .001 | .000 | .103 | 1.922 | .055 |
| a. Dependent Variable: BSDMN Residual | | | | | | |

| **Model Summary** | | | | |
| --- | --- | --- | --- | --- |
| Model | R | R Square | Adjusted R Square | Std. Error of the Estimate |
| 1 | .101^a^ | .010 | .007 | .06128720 |
| a. Predictors: (Constant), New Sleep % Residual | | | | |

| **Coefficients^a^** | | | | | | |
| --- | --- | --- | --- | --- | --- | --- |
| Model | | Unstandardized Coefficients | | Standardized Coefficients | t | Sig. |
|  |  | B | Std. Error | Beta |  |  |
| 1 | (Constant) | .002 | .003 |  | .613 | .540 |
|  | New Sleep % Residual | .001 | .001 | .101 | 1.887 | .060 |
| a. Dependent Variable: BSDAN Residual | | | | | | |

| **Model Summary** | | | | |
| --- | --- | --- | --- | --- |
| Model | R | R Square | Adjusted R Square | Std. Error of the Estimate |
| 1 | .072^a^ | .005 | .002 | .06105031 |
| a. Predictors: (Constant), New Sleep % Residual | | | | |

| **Coefficients^a^** | | | | | | |
| --- | --- | --- | --- | --- | --- | --- |
| Model | | Unstandardized Coefficients | | Standardized Coefficients | t | Sig. |
|  |  | B | Std. Error | Beta |  |  |
| 1 | (Constant) | -7.010E-5 | .003 |  | -.021 | .983 |
|  | New Sleep % Residual | -.001 | .001 | -.072 | -1.341 | .181 |
| a. Dependent Variable: VIS Residual | | | | | | |

### Total Sleep Time (NOTE: Predictive of ECN. Included in main paper)

###

| **Model Summary** | | | | |
| --- | --- | --- | --- | --- |
| Model | R | R Square | Adjusted R Square | Std. Error of the Estimate |
| 1 | .038^a^ | .001 | -.001 | .06083155 |
| a. Predictors: (Constant), New TST Residual | | | | |

| **Coefficients^a^** | | | | | | |
| --- | --- | --- | --- | --- | --- | --- |
| Model | | Unstandardized Coefficients | | Standardized Coefficients | t | Sig. |
|  |  | B | Std. Error | Beta |  |  |
| 1 | (Constant) | .002 | .003 |  | .676 | .500 |
|  | New TST Residual | 4.515E-5 | .000 | .038 | .697 | .486 |
| a. Dependent Variable: DMN Residual | | | | | | |

| **Model Summary** | | | | |
| --- | --- | --- | --- | --- |
| Model | R | R Square | Adjusted R Square | Std. Error of the Estimate |
| 1 | .011^a^ | .000 | -.003 | .02886808 |
| a. Predictors: (Constant), New TST Residual | | | | |

| **Coefficients^a^** | | | | | | |
| --- | --- | --- | --- | --- | --- | --- |
| Model | | Unstandardized Coefficients | | Standardized Coefficients | t | Sig. |
|  |  | B | Std. Error | Beta |  |  |
| 1 | (Constant) | .000 | .002 |  | -.108 | .914 |
|  | New TST Residual | -6.341E-6 | .000 | -.011 | -.206 | .837 |
| a. Dependent Variable: DAN Residual | | | | | | |

| **Model Summary** | | | | |
| --- | --- | --- | --- | --- |
| Model | R | R Square | Adjusted R Square | Std. Error of the Estimate |
| 1 | .055^a^ | .003 | .000 | .08917536 |
| a. Predictors: (Constant), New TST Residual | | | | |

| **Coefficients^a^** | | | | | | |
| --- | --- | --- | --- | --- | --- | --- |
| Model | | Unstandardized Coefficients | | Standardized Coefficients | t | Sig. |
|  |  | B | Std. Error | Beta |  |  |
| 1 | (Constant) | .000 | .005 |  | -.045 | .964 |
|  | New TST Residual | -9.787E-5 | .000 | -.055 | -1.031 | .303 |
| a. Dependent Variable: SAL Residual | | | | | | |

| **Model Summary** | | | | |
| --- | --- | --- | --- | --- |
| Model | R | R Square | Adjusted R Square | Std. Error of the Estimate |
| 1 | .071^a^ | .005 | .002 | .04095339 |
| a. Predictors: (Constant), New TST Residual | | | | |

| **Coefficients^a^** | | | | | | |
| --- | --- | --- | --- | --- | --- | --- |
| Model | | Unstandardized Coefficients | | Standardized Coefficients | t | Sig. |
|  |  | B | Std. Error | Beta |  |  |
| 1 | (Constant) | .002 | .002 |  | .755 | .451 |
|  | New TST Residual | 5.761E-5 | .000 | .071 | 1.322 | .187 |
| a. Dependent Variable: BSDMN Residual | | | | | | |

| **Model Summary** | | | | |
| --- | --- | --- | --- | --- |
| Model | R | R Square | Adjusted R Square | Std. Error of the Estimate |
| 1 | .001^a^ | .000 | -.003 | .06160256 |
| a. Predictors: (Constant), New TST Residual | | | | |

| **Coefficients^a^** | | | | | | |
| --- | --- | --- | --- | --- | --- | --- |
| Model | | Unstandardized Coefficients | | Standardized Coefficients | t | Sig. |
|  |  | B | Std. Error | Beta |  |  |
| 1 | (Constant) | .002 | .003 |  | .610 | .542 |
|  | New TST Residual | 1.367E-6 | .000 | .001 | .021 | .983 |
| a. Dependent Variable: BSDAN Residual | | | | | | |

| **Model Summary** | | | | |
| --- | --- | --- | --- | --- |
| Model | R | R Square | Adjusted R Square | Std. Error of the Estimate |
| 1 | .096^a^ | .009 | .006 | .10250036 |
| a. Predictors: (Constant), New TST Residual | | | | |

| **Coefficients^a^** | | | | | | |
| --- | --- | --- | --- | --- | --- | --- |
| Model | | Unstandardized Coefficients | | Standardized Coefficients | t | Sig. |
|  |  | B | Std. Error | Beta |  |  |
| 1 | (Constant) | .001 | .006 |  | .095 | .924 |
|  | New TST Residual | .000 | .000 | -.096 | -1.783 | .075 |
| a. Dependent Variable: MOT Residual | | | | | | |

| **Model Summary** | | | | |
| --- | --- | --- | --- | --- |
| Model | R | R Square | Adjusted R Square | Std. Error of the Estimate |
| 1 | .130^a^ | .017 | .014 | .06068895 |
| a. Predictors: (Constant), New TST Residual | | | | |

| **Coefficients^a^** | | | | | | |
| --- | --- | --- | --- | --- | --- | --- |
| Model | | Unstandardized Coefficients | | Standardized Coefficients | t | Sig. |
|  |  | B | Std. Error | Beta |  |  |
| 1 | (Constant) | -7.010E-5 | .003 |  | -.022 | .983 |
|  | New TST Residual | .000 | .000 | -.130 | -2.437 | .015 |
| a. Dependent Variable: VIS Residual | | | | | | |

### Wake After Sleep Onset Time (Note: Predictive of Salience and Motor Network. Included in main paper)

| **Model Summary** | | | | |
| --- | --- | --- | --- | --- |
| Model | R | R Square | Adjusted R Square | Std. Error of the Estimate |
| 1 | .035^a^ | .001 | -.002 | .06083612 |
| a. Predictors: (Constant), New WASO time Residual | | | | |

| **Coefficients^a^** | | | | | | |
| --- | --- | --- | --- | --- | --- | --- |
| Model | | Unstandardized Coefficients | | Standardized Coefficients | t | Sig. |
|  |  | B | Std. Error | Beta |  |  |
| 1 | (Constant) | .002 | .003 |  | .675 | .500 |
|  | New WASO time Residual | -7.117E-5 | .000 | -.035 | -.659 | .510 |
| a. Dependent Variable: DMN Residual | | | | | | |

| **Model Summary** | | | | |
| --- | --- | --- | --- | --- |
| Model | R | R Square | Adjusted R Square | Std. Error of the Estimate |
| 1 | .080^a^ | .006 | .003 | .02790248 |
| a. Predictors: (Constant), New WASO time Residual | | | | |

| **Coefficients^a^** | | | | | | |
| --- | --- | --- | --- | --- | --- | --- |
| Model | | Unstandardized Coefficients | | Standardized Coefficients | t | Sig. |
|  |  | B | Std. Error | Beta |  |  |
| 1 | (Constant) | .001 | .001 |  | .334 | .738 |
|  | New WASO time Residual | -7.366E-5 | .000 | -.080 | -1.487 | .138 |
| a. Dependent Variable: ECN Residual | | | | | | |

| **Model Summary** | | | | |
| --- | --- | --- | --- | --- |
| Model | R | R Square | Adjusted R Square | Std. Error of the Estimate |
| 1 | .050^a^ | .002 | .000 | .02883403 |
| a. Predictors: (Constant), New WASO time Residual | | | | |

| **Coefficients^a^** | | | | | | |
| --- | --- | --- | --- | --- | --- | --- |
| Model | | Unstandardized Coefficients | | Standardized Coefficients | t | Sig. |
|  |  | B | Std. Error | Beta |  |  |
| 1 | (Constant) | .000 | .002 |  | -.108 | .914 |
|  | New WASO time Residual | -4.740E-5 | .000 | -.050 | -.926 | .355 |
| a. Dependent Variable: DAN Residual | | | | | | |

| **Variables Entered/Removed^a^** | | | |
| --- | --- | --- | --- |
| Model | Variables Entered | Variables Removed | Method |
| 1 | New WASO time Residual^b^ | . | Enter |
| a. Dependent Variable: BSDMN Residual | | | |
| b. All requested variables entered. | | | |

| **Model Summary** | | | | |
| --- | --- | --- | --- | --- |
| Model | R | R Square | Adjusted R Square | Std. Error of the Estimate |
| 1 | .082^a^ | .007 | .004 | .04092001 |
| a. Predictors: (Constant), New WASO time Residual | | | | |

| **Coefficients^a^** | | | | | | |
| --- | --- | --- | --- | --- | --- | --- |
| Model | | Unstandardized Coefficients | | Standardized Coefficients | t | Sig. |
|  |  | B | Std. Error | Beta |  |  |
| 1 | (Constant) | .002 | .002 |  | .756 | .450 |
|  | New WASO time Residual | .000 | .000 | -.082 | -1.521 | .129 |
| a. Dependent Variable: BSDMN Residual | | | | | | |

| **Model Summary** | | | | |
| --- | --- | --- | --- | --- |
| Model | R | R Square | Adjusted R Square | Std. Error of the Estimate |
| 1 | .105^a^ | .011 | .008 | .06126433 |
| a. Predictors: (Constant), New WASO time Residual | | | | |

| **Coefficients^a^** | | | | | | |
| --- | --- | --- | --- | --- | --- | --- |
| Model | | Unstandardized Coefficients | | Standardized Coefficients | t | Sig. |
|  |  | B | Std. Error | Beta |  |  |
| 1 | (Constant) | .002 | .003 |  | .614 | .540 |
|  | New WASO time Residual | .000 | .000 | -.105 | -1.955 | .051 |
| a. Dependent Variable: BSDAN Residual | | | | | | |

| **Model Summary** | | | | |
| --- | --- | --- | --- | --- |
| Model | R | R Square | Adjusted R Square | Std. Error of the Estimate |
| 1 | .051^a^ | .003 | .000 | .06113094 |
| a. Predictors: (Constant), New WASO time Residual | | | | |

| **Coefficients^a^** | | | | | | |
| --- | --- | --- | --- | --- | --- | --- |
| Model | | Unstandardized Coefficients | | Standardized Coefficients | t | Sig. |
|  |  | B | Std. Error | Beta |  |  |
| 1 | (Constant) | -7.010E-5 | .003 |  | -.021 | .983 |
|  | New WASO time Residual | .000 | .000 | .051 | .940 | .348 |
| a. Dependent Variable: VIS Residual | | | | | | |

### Wake After Sleep Onset Number (Note: Predicitive of ECN, SAL, and MOT networks. Included in main paper)

| **Model Summary** | | | | |
| --- | --- | --- | --- | --- |
| Model | R | R Square | Adjusted R Square | Std. Error of the Estimate |
| 1 | .002^a^ | .000 | -.003 | .06087429 |
| a. Predictors: (Constant), New Waso N Residual | | | | |

| **Coefficients^a^** | | | | | | |
| --- | --- | --- | --- | --- | --- | --- |
| Model | | Unstandardized Coefficients | | Standardized Coefficients | t | Sig. |
|  |  | B | Std. Error | Beta |  |  |
| 1 | (Constant) | .002 | .003 |  | .675 | .500 |
|  | New Waso N Residual | 2.064E-5 | .001 | .002 | .037 | .970 |
| a. Dependent Variable: DMN Residual | | | | | | |

| **Model Summary** | | | | |
| --- | --- | --- | --- | --- |
| Model | R | R Square | Adjusted R Square | Std. Error of the Estimate |
| 1 | .030^a^ | .001 | -.002 | .02885711 |
| a. Predictors: (Constant), New Waso N Residual | | | | |

| **Coefficients^a^** | | | | | | |
| --- | --- | --- | --- | --- | --- | --- |
| Model | | Unstandardized Coefficients | | Standardized Coefficients | t | Sig. |
|  |  | B | Std. Error | Beta |  |  |
| 1 | (Constant) | .000 | .002 |  | -.108 | .914 |
|  | New Waso N Residual | .000 | .000 | -.030 | -.552 | .581 |
| a. Dependent Variable: DAN Residual | | | | | | |

| **Model Summary** | | | | |
| --- | --- | --- | --- | --- |
| Model | R | R Square | Adjusted R Square | Std. Error of the Estimate |
| 1 | .035^a^ | .001 | -.002 | .04103185 |
| a. Predictors: (Constant), New Waso N Residual | | | | |

| **Coefficients^a^** | | | | | | |
| --- | --- | --- | --- | --- | --- | --- |
| Model | | Unstandardized Coefficients | | Standardized Coefficients | t | Sig. |
|  |  | B | Std. Error | Beta |  |  |
| 1 | (Constant) | .002 | .002 |  | .754 | .451 |
|  | New Waso N Residual | .000 | .000 | -.035 | -.650 | .516 |
| a. Dependent Variable: BSDMN Residual | | | | | | |

| **Model Summary** | | | | |
| --- | --- | --- | --- | --- |
| Model | R | R Square | Adjusted R Square | Std. Error of the Estimate |
| 1 | .083^a^ | .007 | .004 | .06138882 |
| a. Predictors: (Constant), New Waso N Residual | | | | |

| **Coefficients^a^** | | | | | | |
| --- | --- | --- | --- | --- | --- | --- |
| Model | | Unstandardized Coefficients | | Standardized Coefficients | t | Sig. |
|  |  | B | Std. Error | Beta |  |  |
| 1 | (Constant) | .002 | .003 |  | .612 | .541 |
|  | New Waso N Residual | -.001 | .001 | -.083 | -1.551 | .122 |
| a. Dependent Variable: BSDAN Residual | | | | | | |

| **Model Summary** | | | | |
| --- | --- | --- | --- | --- |
| Model | R | R Square | Adjusted R Square | Std. Error of the Estimate |
| 1 | .043^a^ | .002 | -.001 | .06115159 |
| a. Predictors: (Constant), New Waso N Residual | | | | |

| **Coefficients^a^** | | | | | | |
| --- | --- | --- | --- | --- | --- | --- |
| Model | | Unstandardized Coefficients | | Standardized Coefficients | t | Sig. |
|  |  | B | Std. Error | Beta |  |  |
| 1 | (Constant) | -7.010E-5 | .003 |  | -.021 | .983 |
|  | New Waso N Residual | .000 | .001 | -.043 | -.806 | .421 |
| a. Dependent Variable: VIS Residual | | | | | | |
